# A Large Scale Gene-Centric Association Study of Lung Function in Newly-Hired Female Cotton Textile Workers with Endotoxin Exposure

**DOI:** 10.1371/journal.pone.0059035

**Published:** 2013-03-19

**Authors:** Ruyang Zhang, Yang Zhao, Minjie Chu, Amar Mehta, Yongyue Wei, Yao Liu, Pengcheng Xun, Jianling Bai, Hao Yu, Li Su, Hongxi Zhang, Zhibin Hu, Hongbing Shen, Feng Chen, David C. Christiani

**Affiliations:** 1 Department of Epidemiology and Biostatistics and Ministry of Education (MOE) Key Lab for Modern Toxicology, School of Public Health, Nanjing Medical University, Nanjing, China; 2 Section of Clinical Epidemiology, Jiangsu Key Laboratory of Cancer Biomarkers, Prevention and Treatment, Cancer Center, Nanjing Medical University, Nanjing, China; 3 Department of Environmental Health, Harvard School of Public Health, Boston, Massachusetts, United States of America; 4 Swiss Tropical and Public Health Institute, Basel, Switzerland; 5 University of Basel, Basel, Switzerland; 6 Department of Epidemiology and Biostatistics, School of Public Health, Indiana University Bloomington, Bloomington, Indiana, United States of America; 7 Putuo District Peoples Hospital, Shanghai Second Medical University, Shanghai, China; 8 Pulmonary and Critical Care Unit, Department of Medicine, Massachusetts General Hospital/Harvard Medical School, Boston, Massachusetts, United States of America; Johns Hopkins University, United States of America

## Abstract

**Background:**

Occupational exposure to endotoxin is associated with decrements in pulmonary function, but how much variation in this association is explained by genetic variants is not well understood.

**Objective:**

We aimed to identify single nucleotide polymorphisms (SNPs) that are associated with the rate of forced expiratory volume in one second (FEV_1_) decline by a large scale genetic association study in newly-hired healthy young female cotton textile workers.

**Methods:**

DNA samples were genotyped using the Illumina Human CVD BeadChip. Change rate in FEV_1_ was modeled as a function of each SNP genotype in linear regression model with covariate adjustment. We controlled the type 1 error in study-wide level by permutation method. The false discovery rate (FDR) and the family-wise error rate (FWER) were set to be 0.10 and 0.15 respectively.

**Results:**

Two SNPs were found to be significant (*P*<6.29×10^−5^), including rs1910047 (*P = *3.07×10^−5^, FDR = 0.0778) and rs9469089 (*P = *6.19×10^−5^, FDR = 0.0967), as well as other eight suggestive (*P*<5×10^−4^) associated SNPs. Gene-gene and gene-environment interactions were also observed, such as rs1910047 and rs1049970 (*P = *0.0418, FDR = 0.0895); rs9469089 and age (*P = *0.0161, FDR = 0.0264). Genetic risk score analysis showed that the more risk loci the subjects carried, the larger the rate of FEV_1_ decline occurred (*P*_trend_ = 3.01×10^−18^). However, the association was different among age subgroups (*P* = 7.11×10^−6^) and endotoxin subgroups (*P* = 1.08×10^−2^). Functional network analysis illustrates potential biological connections of all interacted genes.

**Conclusions:**

Genetic variants together with environmental factors interact to affect the rate of FEV_1_ decline in cotton textile workers.

## Introduction

According to an official statement from the American Thoracic Society (ATS) [1], cigarette smoking is not the sole meaningful cause of chronic obstructive pulmonary disease (COPD). There is also strong evidence suggesting a causal relationship between COPD and occupational exposures (e.g. endotoxin [2]) or genetic syndromes (e.g. *α*_1_-antitrypsin deficiency [3]). Generally released from bacterial lysis, endotoxin is ubiquitous in the airborne environment [4]. High endotoxin levels are observed in cotton textile mills processing cotton. Exposure to cotton dust leads to chronic respiratory disease and excessive loss of pulmonary function [5]–[7]. Recently, genome-wide association studies (GWAS) [8]–[10] and large-scale meta-analysis of GWAS [11]–[13] of COPD also offered mechanistic insight into pulmonary function regulation due to individual genetic heterogeneity (e.g. 6p21.32 region) in non-occupationally exposed populations.

Even for occupationally exposed populations, there is evidence that single nucleotide polymorphisms (SNPs) may play an important role in pulmonary function while taking endotoxin effect into account. Our previous study in 20-year longitudinal cohort suggested that *Tyr113His* and *His139Arg* polymorphisms in microsomal epoxide hydrolase (*mEH*) gene [14], rs1800629 in tumor necrosis factor (*TNF*) gene and rs909253 in lymphotoxin alpha (*LTA*) gene [15], may modify the association between endotoxin exposure and annual decline of FEV_1_ in cotton textile workers, though no statistically significant marginal effects of those polymorphisms were observed.

In this study, we aimed to explore the association between the rate of FEV_1_ decline and genetic heterogeneity in previously unexposed newly-hired young healthy female cotton textile workers entering endotoxin-exposed work areas in yarn preparation based on large scale genomic data. To the best of our knowledge, there is no large scale association study of lung function in occupational settings.

## Materials and Methods

### Ethics Statement

The institutional review boards of the Harvard School of Public Health, the Putuo District People’s Hospital, and the Human Resources Administration of China approved the study. All participants gave written informed consent before the study.

### Study Population

The study population was derived from the Newly-Hired Chinese Textile Workers Study, an intensive, repeated measures prospective cohort study in the cotton textile industry consisting of 384 adults in Shanghai, China. All subjects were self-reported Han Chinese ethnicity, females, and lifelong non-smokers, asymptomatic of cardiopulmonary disease and naive to occupational endotoxin exposure before entering cotton textile mill. Detailed information on subject selection, methods for testing pulmonary function, and exposure assessment has been described previously [16]–[18]. A baseline examination took place in March of 1997, and follow-up examinations occurred at 3 months, 12 months, and 18 months later. Only subjects with DNA samples that met quality assurance requirements for genotyping and analysis (*n* = 301; refer to quality control section for details) were included in this study. Among the 301 subjects, one hundred and sixty-three (54.2%) subjects with average age of 18.4 years (range 16.0–28.8 years) were newly employed, while 138 (45.8%) subjects with average age of 33.1 years (range 16.6–46.7 years) were currently working in converted silk mills. Sixty-two (20.6%) subjects were followed up to 3 months, 42 (14.0%) subjects were followed up to 12 months, and 197 (65.4%) subjects were followed up to 18 months.

### Exposure Assessment and Pulmonary Function Tests

Briefly, airborne cotton dust was sampled with a settled vertical elutriator (General Metalworks Corp., Mequon, WI) fixed in work area using 37 mm PVC filters, and endotoxin concentration was analyzed using chromogenic Limulus amoebocyte lysate assay, as previously described [19]. The workers remained in the same area throughout the observation. We recorded the endotoxin concentration for all work areas at each follow-up. Spirometric measurements were conducted by a trained technician at cotton mills using an 8-liter water-sealed field spirometer (W.E. Collins Co., Braintree, MA) calibrated twice each day with a 3-liter syringe. Forced expiratory spirograms were carried out before and after work shifts on the first day back to work after a two day rest, and each worker performed up to seven trials to produce three acceptable curves [16]. The present analysis focused on change rate in pre-shift FEV_1_ over the 18 month period of follow-up.

### DNA Isolation and SNP Genotyping

DNA samples were extracted from whole blood using a DNA purification kit (Gentra Systems, Inc., Research Triangle, NC). Of the 384 DNA samples collected from subjects who participated in the baseline examination, 55 samples were excluded due to inadequate DNA content or low DNA quality. Genotyping was performed using the Illumina HumanCVD Beadchip (IBC array) by laboratory personnel without the knowledge of phenotype information. The IBC array incorporates about 50,000 SNPs to efficiently capture genetic diversity across >2,000 genic regions related to cardiovascular, inflammatory and metabolic phenotypes. Genetic variation within the majority of these regions is captured at density equal to or greater than that afforded by genome-wide genotyping products [20]. Of the 329 samples that were sent for genotyping, 312 were genotyped successfully.

### Quality Control

We performed systematic quality control procedures to filter both unqualified samples and SNPs before the association analysis. Briefly, SNPs were removed for a total of 49,094 genotyped ones if they were located in non-autosomal chromosomes (1,126), had a call rate ≤95% (954), minor allele frequency (MAF) <0.05 (19,357), *P*<0.001 for Hardy-Weinberg equilibrium (HWE) test (46). For these 312 samples, one subject was removed with a genotyping call rate <95%. None was ambiguous for genetic sex. Seven subjects failed the cryptic relatedness check. We also detected outliers (three individuals were excluded) and population stratification using EIGENSTRAT 4.2 [21], a method based on principal component analysis. As a result, a total of 301 subjects and 27,611 SNPs were left for subsequent analysis. Quality control procedures were implemented in PLINK (version 1.07) [22].

### Statistical Methods

As presented in **Figure S1 in File S1**, the FEV_1_ declines linearly with increased exposure time, but the rate of FEV_1_ decline varies among individuals. We aimed to evaluate the association between SNP and the rate of FEV_1_ decline. Firstly, we estimated the rate of monthly FEV_1_ declines for each individual in a linear regression model through **Equation 1**. For the *i* ^th^ individual, *FEV*_1*ij*_ is the observed FEV_1_ value at different months (*month_j_* = 0, 3, 12 and 18), *u*_0*i*_ is baseline FEV_1_ and *e_ij_* is the random error at *j* ^th^ months. The estimated slope (*ES_i_*) reflected the rate of FEV_1_ decline as a function of month for each individual. The mean and median *R* ^2^ of the linear model for all 301 subjects are 0.63 and 0.73 respectively indicating the present model is not bad to describe the monthly decline rate of FEV_1_. *ES_i_* was then used as the outcome in the following association analysis.
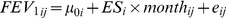
(1)


Secondly, we assessed the associations between *ES* and each SNP by fitting multiple-variable linear regression models (**Equation 2**) adjusted for height, age, FEV_1_ at baseline and the average level of log-transformed endotoxin exposure. In **Equation 2**, *e_i_* is the random error for *i* ^th^ individual. Two genetic models (dominant and additive) of inheritance for each SNP were considered respectively. In additive model, SNPs with low frequency (≤5%) of rare homozygous were excluded for lack of statistical robustness [23].
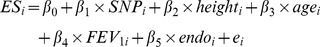
(2)


The bootstrap re-sampling analysis was then performed to control the false positive discovery for significant SNPs [24]. We generated 2,000 bootstrap samples, and SNPs were tested 2,000 times respectively. The results are not likely to be falsely positive if the SNP has more than 1,600 times (80%) bootstrap *P* value less than the predefined threshold (e.g. 0.005 for 10 SNPs).

Gene-gene and gene-environment interactions were tested using **Equation 3**. Here, *β_A_* and *β_B_* are the main effects of factor *A* and *B*, respectively. *β_AB_* is the interaction.
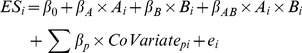
(3)


We also constructed a genetic risk score (GRS) by counting the number of risk genotypes of top 10 SNPs that the individuals carried [25]. The association between the GRS and the rate of FEV_1_ decline was also tested in multiple-variable linear regression models to evaluate the join effects of multiple genetic factors. Additionally, stratification analysis of GRS by age and endotoxin level was performed.

Due to the linkage disequilibrium (LD) structure among SNPs, a standard Bonferroni correction would yield a significance level of approximately 1.81×10^−6^, which is very conservative. And, the Human CVD array has a dense gene-centric design, similar studies have used a less stringent level around 1×10^−4^ previously [26], [27]. In this exploratory study, we controlled the false discovery rate (FDR) and the family-wise error rate (FWER) in study-wide level by permutation method (see details in **Appendix S1**). The FDR was set to be 0.10, meanwhile the FWER were set to be 0.15 (corresponding threshold for each SNP was 6.29×10^−5^). We imputed un-genotyped SNPs using Minimac software [28] based on LD information from hg/19 1000 Genomes database (with ASN as the reference set, released November 2010). Gene functional network was built using MetaCore™ online software [29] (GeneGo, Inc., Carlsbad, CA) to illustrate potential biological connections of interacted genes identified in this study. Statistical analysis and data management were carried out through: PLINK (Version 1.07) [22] and R software (version 2.14.0; The R Foundation for Statistical Computing). Manhattan plots were generated using Haploview (version 4.2) [30].

## Results

The mean decline of FEV_1_ for the overall 301 subjects was 6.79±25.09 ml per-month. Individuals were classified to different subgroups by age, height, FEV_1_ at baseline and endotoxin level respectively and the rate of FEV_1_ decline (*ES*) in each subgroup was presented in **Table 1**. The large standard deviation (SD) of *ES* reflects a large individual variation of *ES* among subjects, which probably be caused by different environmental exposure, demographic characteristics or genetic background. We fitted a multi-variable linear regression model (**Equation 2**) and predicted the *ES* for each subject. The SD of the predictive *ES* was presented in **Table S1 in File S1**. When deducted the effects of non-genetic factors, the SD of predictive *ES* ranges from ∼4 to ∼6. We assumed that the genetic factors might contribute to the remained variation of *ES*.

**Table 1:** The mean and standard deviation of the rate of FEV_1_ decline (*ES*) in different subgroups.

Subgroup	Mean ± SD (ml/month) of the rate of FEV_1_ decline (*n*)					
	Age^a^ <18years		18≤ Age^a^ <25years		Age^a^ ≥25years	
Height^b^						
Low	0.65±24.53	30	−12.00±45.37	40	−8.47±14.65	44
High	−1.73±23.47	43	−2.95±23.68	67	−12.67±9.08	77
FEV_1_ b						
Low	−0.60±13.16	31	−5.13±46.24	37	−10.38±13.35	80
High	−0.86±29.43	42	−7.00±24.23	70	−12.72±6.23	41
Endotoxin^b^						
Low	−8.66±29.19	15	−5.18±59.18	25	−11.69±8.41	107
High	1.29±22.00	58	−4.70±20.11	82	−15.86±36.29	14

a Subjects with age ≥ years were divided into two additional groups according median (25 years);

b Subjects were divided into two groups according to median (160 cm, 2630 ml and 163 EU/m^3^ for height, FEV_1_ and endotoxin respectively);

### Results of Individual SNP Analysis

In additive model, the frequency of rare homozygosity of the top-10 SNPs was less than 2.5% (data not shown) and none was significant. Thus, only SNPs identified by the dominant model are presented (**Table 2**, **Tables S2 and S3 in File S1**). Two risk SNPs (rs1910047 and rs9469089) were significant with *P*<6.29×10^−5^. Individuals carrying the TT genotype of rs1910047 had average 4.32 ml decline of FEV_1_ per-month, while those carrying the TA or AA genotypes had 18.32 ml decline of FEV_1_ per-month. After adjustment of covariates, individuals carrying the AA genotype had more rapid FEV_1_ decline (15.17 ml/moth) compared with those carrying the TA or AA genotype (*P* = 3.70×10^−5^, FDR = 0.0778). For rs9469089, individuals carrying the GC or CC genotype also had more rapid FEV_1_ decline (12.50 ml/month) than those carrying the GG genotype (*P* = 6.19×10^−5^, FDR = 0.0967).

**Table 2:** Summary of results of the association study for top-10 SNPs using dominant model.

SNP	Genotype	*N*	Mean± SD a	*β* (SE) b	*P* b	Proportion c
**rs1910047**	TT	248	−4.32±21.86	−15.17(3.62)	3.70×10^−5^	89.25%
	TA+AA	53	−18.32±34.61			
**rs9469089**	GG	217	−3.07±23.35	−12.50(3.08)	6.19×10^−5^	96.35%
	GC+CC	83	−16.69±26.98			
rs32588	TT	268	−8.71±23.60	17.21(4.39)	1.11×10^−4^	85.50%
	TC+CC	33	8.84±31.18			
rs4855881	AA	257	−9.08±22.28	14.83(3.92)	1.84×10^−4^	83.35%
	AG+GG	44	6.60±34.96			
rs10515978	AA	138	−12.59±23.71	10.09(2.77)	3.16×10^−4^	90.75%
	AG+GG	163	−1.88±25.25			
rs601675	AA	160	−2.19±18.74	−10.14(2.79)	3.32×10^−4^	85.80%
	AG+GG	141	−12.00±29.98			
rs11761231	AA	167	−11.54±21.07	10.05(2.77)	3.35×10^−4^	89.60%
	AG+GG	134	−0.86±28.32			
rs10129426	CC	100	−13.44±27.26	10.52(2.93)	3.88×10^−4^	82.85%
	CT+TT	201	−3.38±23.31			
rs10201627	GG	241	−4.23±23.05	−12.35(3.47)	4.41×10^−4^	85.45%
	GT+TT	60	−17.05±30.10			
rs1049970	CC	194	−2.99±23.16	−10.30(2.91)	4.63×10^−4^	86.60%
	CT+TT	106	−14.12±26.77			

a Mean and standard deviation of the rate of FEV_1_ decline (ml/month);

b Coefficient, standard error and *P* value to SNP in linear regression model adjusted for height, age and FEV_1_ at baseline, and average log transformed endotoxin level;

c Proportions of *P* value ≤0.005 in 2,000 bootstrap samples.

Eight SNPs (rs32588, rs4855881, rs10515978, rs601675, rs11761231, rs10129426, rs10201627 and rs1049970) with *P*<5×10^−4^ were considered suggestive for accelerated changes of FEV_1_. Two SNPs (rs32586 and rs32589) are in perfect LD with rs32588 (*R* ^2^ = 1.00). rs3749073 is also in high LD with rs10201627 (*R* ^2^ = 0.98) (**Table S4 in File S1**). The bootstrap re-sampling analysis was then performed for the top-10 SNPs to internally validate the results. All top-10 SNPs had a bootstrap *P* value<0.005 more than 1,600 times out of 2,000 bootstrap samples (over 80% significant), indicating that those SNPs were not likely to be false-positive results.

Manhattan plot using *P* values derived from the dominant model are presented in **Figure S2 in File S1**. A small genomic control inflation factor (*λ*) of 1.026 indicated little inflation of the large scale genotyping study results due to population stratification (**Figure S3 in File S1**). We again did a sensitivity analysis with further adjustment of first eight PCs generated by EIGENSTRAT 4.2. However, none PCs was significant in the model (*P* values range from 0.24 to 0.82). As presented in **Table S5 in File S1**, the results do not vary much whenever PCs adjusted or not.

After imputation, a cluster of adjacent SNPs in high or low LD with targeted SNP were observed with *P* value from 10^−5^ to 10^−3^ (**Appendix S2** and **Table S6 in File S2**). We also tested those 10 SNPs in additive model (**Table S7 in File S1**). All SNPs were significant at 0.005 level, except for rs10515978 (*P* = 0.0159). Considering 73 subjects are young women who may still have been in the lung function growth period of their development, we did another sensitivity analysis only in those subjects with age ≥18 years. As a result, the effects of SNPs do not vary much between all samples and only adult samples (**Table S8 in File S1**).

### Results of Gene-gene and Gene-environment Interaction Analysis

**Figure 1:**
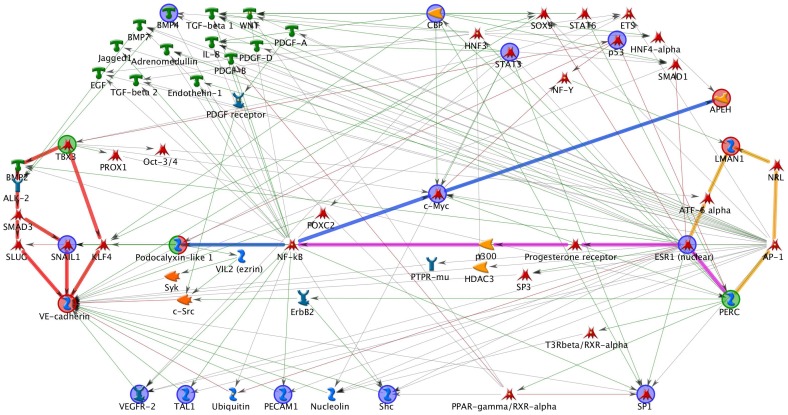
The functional network based on four pairs of gene-gene interactions identified in this study. Four gene-gene interaction functional paths were highlighted, including ***TBX3*** - *VE_cadherin* (***CDH5***), *PERC* (***PPARGC1B***) - ***LMAN1*** **,** *PERC* (***PPARGC1B***) - *Podocalyxin_like 1* (***PODXL***) and *Podocalyxin_like 1* (***PODXL***) - ***APEH***.

We tested all pair-wise gene-gene interactions for these ten SNPs, and interactions with *P* value <0.05 and FDR <0.10 were presented in **Table 3**. rs1910047 which had significant marginal effect had synergistic interaction with rs1049970 (*P = *0.0418, FDR = 0.0895). The other three interactions which composed of four SNPs, also showed synergy between SNPs. The interaction *P* values (FDRs) were 0.0051 (0.0313), 0.0052 (0.0313) and 0.0095 (0.0390) for rs10515978_rs32588, rs32588_ rs11761231 and rs11761231_ rs4855881 respectively. We mapped these interacted SNPs to their nearest genes (**Table S3 in File S1**), and built network (**Figure 1**) using the six genes (*TBX3, CDH5, PPARGC1B, LMAN1, PODXL* and *APEH*). There are four highlighted functional paths corresponding to four pairs of gene-gene interactions: ***TBX3*** - *KLF4* - *VE_cadherin* (***CDH5***); *PERC* (***PPARGC1B***) - *ESR1*(*nuclear*) - *ATF_6 alpha* - ***LMAN1***; *PERC* (***PPARGC1B***) - *ESR1*(*nuclear*) - *Progesterone receptor* - *p300* - *NF_kB* - *Podocalyxin_like 1* (***PODXL***); *Podocalyxin_like 1* (***PODXL***) - *NF_kB* - *c_Myc* - ***APEH***. The information of all 63 nodes in the network is given in **Appendix S3**. Fourteen nodes (*Ubiquitin*, *AP_1* and other twelve blue-circle marked ones) directly connect with the four functional paths and form the gene-gene interaction functional network.

**Table 3:** The results of gene-gene interaction analysis for top-10 SNPs (*P*<0.05).

Gene1^a^	Gene2^a^	*N*	Mean± SD^b^			*β* (SE)^c^	*P* c	*P_GG_* d	FDR^e^
**rs1910047**	rs1049970							4.18×10^−2^	0.0895
TT	CC	159	−1.39±23.13			Ref.			
TT	CT+TT	88	−10.05±17.89			−7.69(3.09)	1.35×10^−2^		
TA+AA	CC	35	−10.25±22.17			−9.79(4.36)	2.56×10^−2^		
TA+AA	CT+TT	18	−34.00±47.81			−32.59(5.73)	3.19×10^−8^		
rs10515978	rs32588							5.09×10^−3^	0.0313
AA	TT	123	−13.12±24.95			Ref.			
AA	TC+CC	15	−8.20±7.51			4.11(6.29)	5.14×10^−1^		
AG+GG	TT	145	−4.97±21.79			7.44(2.83)	8.96×10^−3^		
AG+GG	TC+CC	18	23.04±36.25			35.68(5.83)	2.96×10^−9^		
rs11761231	rs32588							5.16×10^−3^	0.0313
AA	TT	150	−12.38±21.74			Ref.			
AA	TC+CC	17	−4.13±11.92			5.01(5.96)	4.02×10^−1^		
AG+GG	TT	118	−4.04±25.10			7.01(2.85)	1.43×10^−2^		
AG+GG	TC+CC	16	22.62±39.11			36.34(6.06)	6.01×10^−9^		
rs11761231	rs4855881							9.51×10^−3^	0.0390
AA	AA	144	−12.14±21.75			Ref.			
AA	AG+GG	23	−7.80±16.14			5.15(5.22)	3.25×10^−1^		
AG+GG	AA	113	−5.18±22.45			6.90(2.90)	1.81×10^−2^		
AG+GG	AG+GG	21	22.37±42.90			31.87(5.44)	1.27×10^−8^		

a rs1910047 (*TBX3*), rs1049970 (*CDH5*), rs10515978 (*LMAN1*), rs32588 (*PPARGC1B*) and rs4855881 (*APEH*);

b Mean and stand deviation of the rate of FEV_1_ decline (ml/month);

c Coefficient, stand error, *P* value were acquired from linear regression model adjusted for height, age and FEV_1_ at baseline, and average log transformed endotoxin level.

d *P* value for gene-gene interaction.

e FDR q-value calculated based on all pair-wise (45 pairs) *P* values of top-10 SNPs.

From the results of gene-environment interaction analysis (**Table 4**), three SNPs (rs9469089, rs1049970 and rs10201627) with FDR <0.10 exhibited interaction with age (*P = *0.0161, FDR = 0.0264; *P = *0.0206, FDR = 0.0264 and *P = *0.0356, FDR = 0.0308 respectively); rs4855881 had interaction with endotoxin level (*P = *0.0454, FDR = 0.4086).

**Table 4:** The results of gene-environment interaction analysis for top-10 SNPs (*P*<0.05).

Gene^a^	Environment	*N*	Mean±SD^b^	*β* (SE)^c^	*P* c	*P_GE_* d	FDR^e^
**rs9469089**	Age (years)					0.0161	0.0264
GG	<18	53	3.16±25.29	Ref.			
GG	18 ∼ 25	80	0.81±28.47	−2.55(4.38)	0.5607		
GG	>25	84	−10.71±12.20	−23.80(10.91)	0.0300		
GC+CC	<18	20	−11.10±15.35	−13.23(6.17)	0.0329		
GC+CC	18 ∼ 25	26	−22.69±40.84	−25.84(5.85)	<0.0001		
GC+CC	>25	37	−15.49±18.17	−28.43(11.53)	0.0143		
rs1049970	Age (years)					0.0206	0.0264
CC	<18	47	2.03±23.22	Ref.			
CC	18 ∼ 25	78	0.78±28.14	−2.63(4.55)	0.5644		
CC	>25	69	−10.67±12.94	−22.43(11.30)	0.0481		
CT+TT	<18	25	−7.36±23.50	−11.05(5.89)	0.0618		
CT+TT	18 ∼ 25	29	−19.86±40.90	−21.77(5.83)	0.0002		
CT+TT	>25	52	−14.17±16.02	−25.78(11.20)	0.0220		
rs10201627	Age (years)					0.0356	0.0308
GG	<18	60	0.62±25.41	Ref.			
GG	18 ∼ 25	87	0.44±26.17	−0.40(4.18)	0.9247		
GG	>25	94	−11.65±15.42	−19.86(10.84)	0.0679		
GT+TT	<18	13	−7.09±12.84	−9.39(7.23)	0.1951		
GT+TT	18 ∼ 25	20	−27.66±48.70	−28.07(6.25)	<0.0001		
GT+TT	>25	27	−13.98±10.02	−22.06(11.57)	0.0576		
rs4855881	Endotoxin (EU/m^3^)					0.0454	0.4086
AA	<163	132	−12.90±22.51	Ref.			
AA	≥163	125	−5.04±21.40	1.46(5.42)	0.7876		
AG+GG	<163	15	12.82±45.55	27.98(6.51)	<0.0001		
AG+GG	≥163	29	3.38±28.41	8.97(6.64)	0.1777		

a rs9469089 (*RNF5*), rs1049970 (*CDH5*), rs10201627 (*GPR55*) and rs4855881 (*APEH*);

b Mean and stand deviation of the rate of FEV_1_ decline (ml/month);

c Coefficient, stand error, *P* value were acquired from linear regression model adjusted for height, age and FEV_1_ at baseline, and average log transformed endotoxin level.

d *P* value for gene-environment interaction.

e FDR q-value calculated based on top-10 SNPs (10 pairs for Age-SNP interactions and 10 pairs for SNP-Endotoxin interactions).

### Results of Genetic Risk Score Analysis

**Figure 2:**
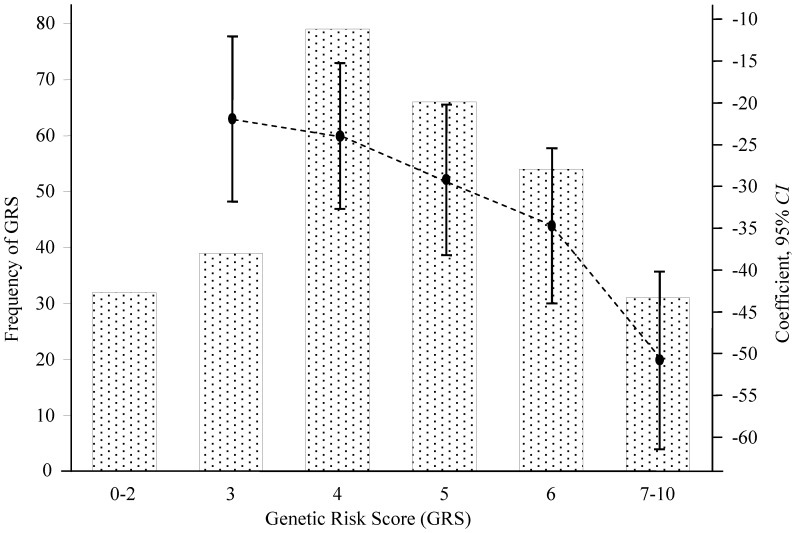
The frequency distribution of genetic risk score (GRS) and the coefficient and 95% *CI* to genetic risk score in linear regression model adjusted for height, age and FEV_1_ at baseline, and average log transformed endotoxin level. The more risk loci the subjects carried, the larger the rate of FEV_1_ decline occurred (*P*_trend_ = 3.01×10^−18^).

**Figure 3:**
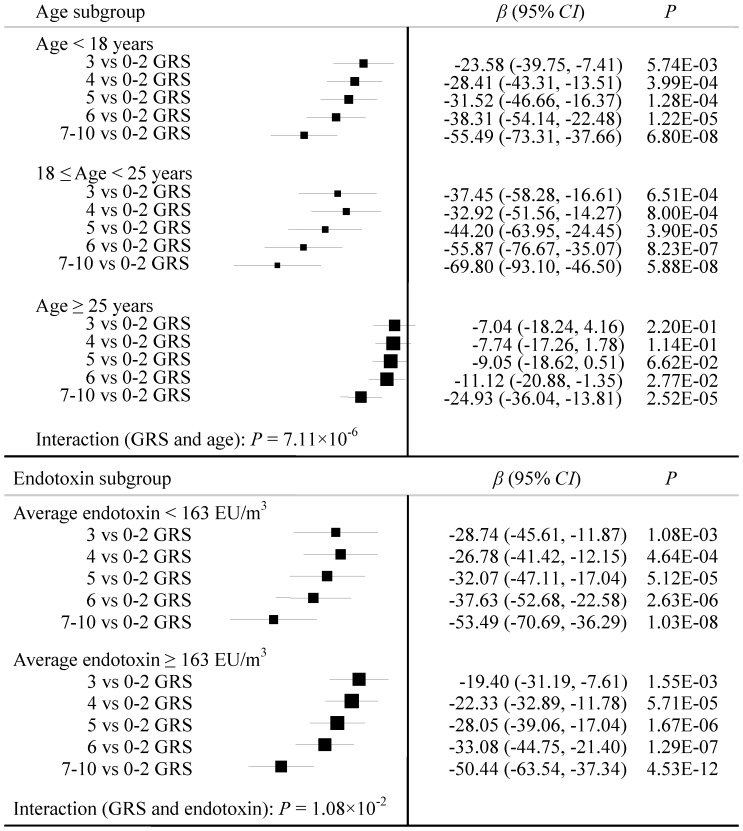
Stratification analysis of the associations between the rate of FEV_1_ decline and the genetic risk score (GRS) of top-10 SNPs. Each box and horizontal line represents the Coefficient and 95% *CI* derived from the linear regression model adjusted for height, age and FEV_1_ at baseline, and average log transformed endotoxin level. *P* values of interaction between GRS and age/endotoxin were acquired from linear regression model with covariates adjustment.

As presented in **Figure 2** and **Table S9 in File S1**, the more risk loci the subjects carried, the larger the rate of FEV_1_ decline occurred (*P*_trend_ = 3.01×10^−18^). Bootstrap re-sampling analysis showed that bootstrap *P*_trend_ was less than 1×10^−15^ with 92.75% of 2,000 bootstrap samples. Additionally, we performed stratification analysis of GRS by age and endotoxin level (**Table S10 in File S1**). Though, the rate of FEV_1_ decline became larger with the increase of GRS, the association differed among age subgroups or endotoxin subgroups, which indicated that there were interactions between GRS and age (*P* = 7.11×10^−6^) or GRS and endotoxin (*P* = 1.08×10^−2^) adjusted for other covariates (**Figure 3**).

## Discussion

Host genetic factors probably influence susceptibility to the rate of FEV_1_ decline after occupational exposure to endotoxin. This exploratory study is the first to provide evidence that genetic factors may play an important role in determining the rate of FEV_1_ decline using a large scale gene-centric aproach. We found that two SNPs (rs1910047 and rs9469089) were significantly associated with the rate of FEV_1_ decline, as well as eight suggestive associated SNPs. Further analysis indicated that potential gene-gene interaction and gene-environment interaction were ubiquitous in the genetic architecture of complex traits.

rs1910047 is located at 12p24.21, upstream of two genes: T-box 5 (*TBX5*, 280 kb upstream) and T-box 3 (*TBX3*, 7 kb upstream). T-box genes encode transcription factors that can mediate the production of branching signals by the lung mesenchyme in the embryonic mouse lung. *TBX4*;*TBX5* double heterozygous mutants showed decreased lung branching, indicating that T-box genes may play an important role in the process of lung growth [31], [32]. In addition, the *Tbx5* was reported to be associated with asthma susceptibility in the developing lung in rat models [33]. Thus, it is biological plausible that SNP variation may result in aberrant activity of the *TBX5* gene, which may affect lung function, accelerating FEV_1_ decline. *TBX3* is a downstream target of Wnt/beta-catenin pathway, which is a key regulator in cell proliferation and differentiation [34]. Recent evidence has suggested that *TBX3* is over-expressed in a number of cancers including lung cancer [34], as well as in transformed lung fibroblast cells [35].

rs9469089 is located at 6p21.32, within the first intron of *RNF5* (encoding membrane-bound ubiquitin ligase). *RNF5* controls the membrane fraction of ATG4B and limits LC3 (ATG8) processing, aberrant of which may limit basal levels of autophagy and influence susceptibility to bacterial infection [36]. Previous studies have collectively suggested that *RNF5* negatively regulates the virus-triggered immune response [37], [38]. Besides, rs9469089 is about 2 kb downstream of advanced glycosylation end product-specific receptor (*AGER*), which is consistently reported to be associated with lung function [11]–[13].

rs1049970 which interacted with rs1910047 is a missense variant of the cadherin 5, type 2 (*CDH5*) gene that may play an important role in endothelial cell biology through control of the cohesion and organization of the intercellular junctions. Deficiency or truncation of Cdh5 induced endothelial apoptosis and abolished transmission of the endothelial survival signal in a mouse model [39]. Both two SNPs are involved in cell stability, including proliferation, differentiation and apoptosis. Thus, it is biologically conceivable that these two SNPs, or those in LD with them, may directly regulate targeted genes, together resulting in more rapid FEV_1_ decline.

It is noteworthy that four SNPs interacted with each other adjacently, producing three pairs of end-to-end interactions in this study (rs10515978_rs32588, rs32588_rs11761231 and rs11761231_rs4855881). rs10515978 is within the intron of lectin, mannose-binding, 1 (*LMAN1*), mutational inactivation of which was a frequent and early event potentially contributing to colorectal tumorigenesis [40]. rs32588 is within the exon of peroxisome proliferator-activated receptor gamma, coactivator 1 beta (*PPARGC1B*), which was associated with airway hyperreactivity in asthmatic patients [41]. rs11761231 was reported to be associated with rheumatoid arthritis in Caucasians [42]–[44]. rs4855881 is located at 3p21.31, within the intron of N-acylaminoacyl-peptide hydrolase (*APEH*). Deletions of which are commonly at the 3p region, and have been found in various types of carcinomas, including lung cancer [45].

Functional network analysis reveals potential biological connections among these four pairs interacted genes. Fourteen nodes as well as other ones directly or indirectly connect to the four functional paths and form the network. Among the fourteen key nodes, genetic variation in the gene encoding p53 can impair the response to cell damage and increase the loss of alveolar epithelial cells [46]. Also, it’s reported that *p53* may modify the effect of diminishing PM**10** exposure on lung function decline [47]. Vascular endothelial growth factor receptor (VEGFR) was reported to mediate the anti-inflammatory, anti-protease, and anti-apoptosis effects of the lung, hence contribute to an attenuation of emphysema and destructive pulmonary function in COPD [48]. Signal transducer and activator of transcription 3 (Stat3) plays an essential role in the pathogenesis of IgG immune complex (IC)-induced acute lung injury [49]. Beoseanrceh morphogenetic protein 4 (BMP4) plays a potential regulatory role of lung fibroblast function [50]. Combined with our gene-gene interaction results, it is possible that the interaction among genes is associated with the rate of FEV_1_ decline through some possible functional paths.

rs10129426 is about 4 kb downstream of BCL2-associated athanogene 5 (*BAG5*), which encodes anti-apoptotic protein that functions through interactions with a variety of cell apoptosis and growth related proteins [51]. According to the UCSC and TFSearch [52] databases, polymorphisms of this gene may influence transcription factor binding (GATA-binding factor 2) [53].

rs10201627 which is located in the micro-RNA binding sites of the 3′-UTR of G protein-coupled receptor 55 (*GPR55*), was predicted to affect miR-26b binding. And it was found that expression of miR-26b was down-regulated in lung cancer tissues [54]. Moreover, rs3749073, as the sole non-synonymous polymorphism (Gly195Val) in the *GPR55*, is in high LD (*r* ^2^ = 0.98) with rs10201627. It was predicted that *GPR55* with Val195 appeared to induce less phosphorylation of extracellular signal-regulated kinase (ERK) than Gly195 [55]. It was also reported that *GPR55* promoted cancer cell proliferation by ERK [56].

Actually, among these top ten SNPs, five SNPs (rs1910047, rs1049970, rs4855881, rs10129426 and rs10201627) are related to cell proliferation or apoptosis and three SNPs (rs9469089, rs32588 and rs11761231) are related to the immune inflammatory response.

We then again investigated the marginal effects of rs1051740 (*Tyr113His*), rs1051741, which was in high LD with rs2234922 (*His139Arg*, not genotyped in our chip), rs1800629 and rs909253 in this study according to our previous studies of long-term pulmonary function decline [14], [15]. None of them reached the statistical significance level. However, rs1051740, rs1051741 and rs909253 could still be significant modifiers in the association between endotoxin exposure and the rate of FEV_1_ decline. The effect of endotoxin exposure was different among subjects carrying different genotypes of SNP. The *P* values of heterogeneity for these three SNPs were 4.83×10^−6^, 6.07×10^−3^ and 1.04×10^−2^ respectively. Although, the subjects in this current study were all newly-hired young healthy female cotton workers, and therefore different from those prospectively followed for 20 years in our previous studies, the results still support what we reported previously. Additionally, four gene-gene interactions and four gene-environment interactions in this study again provided evidence that multiple factors might contribute to the rate of FEV_1_ decline interactively.

Overall, the more risk loci carried by the cotton worker, the larger the rate of FEV_1_ decline over 18 months. However, younger workers (age <25 years) had more accelerated decline per-month than relatively older ones (age ≥25 years) when GRS increased. Moreover, for those carrying same number of risk loci, younger workers had more rapid decline of FEV_1_ than relatively older ones. This provided evidence that endotoxin exposure tended to cause more serious damage to lung function of young workers, even with same genetic background.

Because all the workers of the cohort were lifelong young female non-smokers with relatively pure occupational exposure to endotoxin, this population provided a unique opportunity to observe gene effect to the rate of FEV_1_ decline when adjusted environmental effect. The results contribute scientific support to do further functional studies of those genes in relation to the pathogenesis of endotoxin-related airways disease.

However, it is important to recognize the limitations of our study. Firstly, there is no similar external population to validate the observed SNP associations. Although we have performed internal statistical validation by bootstrap re-sampling procedures to minimize false positive discovery, we could not exclude the possibility of false discoveries among our findings. Besides, we controlled both FDR and FWER in study-wide level (0.10 and 0.15 respectively), which guaranteed the confidence of what we found. Secondly, the sample size was not large enough to detect some SNPs with modest effects. Thus, study with enlarged sample size were warranted. Finally, airborne endotoxin concentrations were estimated from sampling airborne cotton dust at fixed positions in work areas, rather than from sampling the air in the personal breathing zone of each participant, since there is no personal sampling technology developed for these kinds of exposure. The lack of personal air sampling data may be a possible source of exposure misclassification for this study. Thus, we used average of repeated measurement of endotoxin exposure level for each subject.

In summary, we performed the first large scale exploratory study of genetic variation in the rate of FEV_1_ decline for newly-hired young healthy female cotton textile workers exposed to endotoxin. Genetic variants that play a role in immune inflammatory response, cell proliferation and cell apotosis, together with environmental factors interact to affect the rate of FEV_1_ decline after initiation of exposure and this decrement happens over short time. Additional replication and gene function studies are necessary to confirm what we found.

## Supporting Information

File S1 **Supplementary tables (Table S1–S5, S7–S10) and figures (Figure S1–S3).**
(DOCX)

File S2 **The association results of imputed SNPs and genotyped SNPs 500 kb around targeted SNPs (Table S6).**
(XLSX)

Appendix S1 **Description of the FWER and FDR controlling procedure.**
(DOCX)

Appendix S2 **Regional plot of the top-10 SNP using** ***P*** **values derived from multi-variable linear regression model adjusted for height, age and FEV1 at baseline, and average log transformed endotoxin level.**
(PDF)

Appendix S3 **Sixty-three nodes in the gene-gene interaction functional network.**
(DOCX)
