# Supplementary material for: A Large Scale Gene-Centric Association Study of Lung Function in Newly-Hired Female Cotton Textile Workers with Endotoxin Exposure
Source: PLoS One. 2013 Mar 19;8(3):e59035. doi: 10.1371/journal.pone.0059035 (PMC3602449; doi:10.1371/journal.pone.0059035)
Supplement: File S1 — Supplementary tables (Table S1–S5, S7–S10) and figures (Figure S1–S3). (DOCX) [file pone.0059035.s001.docx]

**Table S1**. The mean and standard deviation of the predictive *ES* in different subgroups.

| Subgroup | Mean ± SD (ml/month) of the predictive *ES* (*n*) | | | | | | | | | | | |
| --- | --- | --- | --- | --- | --- | --- | --- | --- | --- | --- | --- | --- |
|  | Age ^a^ < 18 years | | | | 18 ≤ Age ^a^ < 25 years | | | | Age ^a^ ≥ 25 years | | | |
| Height ^b^ |  |  |  |  |  |  |  |  |  |  |  |  |
| Low | -5.68 | ± | **5.09** | 30 | -5.72 | ± | **5.78** | 40 | -13.82 | ± | **5.71** | 44 |
| High | -0.12 | ± | **4.78** | 43 | -3.72 | ± | **4.53** | 67 | -10.14 | ± | **5.49** | 77 |
| FEV_1_ ^b^ |  |  |  |  |  |  |  |  |  |  |  |  |
| Low | -0.45 | ± | **6.05** | 31 | -1.59 | ± | **5.44** | 37 | -9.70 | ± | **4.63** | 80 |
| High | -3.85 | ± | **4.83** | 42 | -5.99 | ± | **4.22** | 70 | -14.95 | ± | **6.39** | 41 |
| Endotoxin ^b^ |  |  |  |  |  |  |  |  |  |  |  |  |
| Low | -6.57 | ± | **3.96** | 15 | -5.79 | ± | **6.49** | 25 | -11.69 | ± | **5.87** | 107 |
| High | -1.33 | ± | **5.48** | 58 | -4.07 | ± | **4.58** | 82 | -9.84 | ± | **5.36** | 14 |

a: Subjects with age ≥ years were divided into two additional groups according median (25 years); b: Subjects were divided into two groups according to median (160 cm, 2630 ml and 163 EU/m^3^ for height, FEV_1_ and endotoxin respectively);

**Table S2**. The FDR q-value of top-10 SNPs determined by permutation method.

| SNP | MLRM ^a^ *P* value | Permutation ^b^ *P* value | FDR q-value |
| --- | --- | --- | --- |
| **rs1910047** | **3.70×10^-5^** | **2.83×10^-6^** | **0.0778** |
| **rs9469089** | **6.19×10^-5^** | **7.02×10^-6^** | **0.0967** |
| rs32588 | 1.11×10^-4^ | 2.18×10^-5^ | 0.1201 |
| rs4855881 | 1.84×10^-4^ | 5.83×10^-4^ | 0.2523 |
| rs10515978 | 3.16×10^-4^ | 1.48×10^-4^ | 0.4199 |
| rs601675 | 3.32×10^-4^ | 1.61×10^-4^ | 0.4346 |
| rs11761231 | 3.35×10^-4^ | 1.63×10^-4^ | 0.4368 |
| rs10129426 | 3.88×10^-4^ | 2.02×10^-4^ | 0.4750 |
| rs10201627 | 4.41×10^-4^ | 2.43×10^-4^ | 0.5052 |
| rs1049970 | 4.63×10^-4^ | 2.61×10^-4^ | 0.5162 |

a: Multi-variable linear regression model including height, age and FEV_1_ at baseline, and average log transformed endotoxin level as covariates.

b: Permutation with 5,000 times.

**Table S3.** Descriptions for top-10 SNPs identified using dominant model.

| CHR | SNP | Position | Region | M/m ^a^ | MAF | *P*_HWE_ | Nearest Gene |
| --- | --- | --- | --- | --- | --- | --- | --- |
| 12 | **rs1910047** | 113613038 | q24.21 | T/A | 0.0947 | 0.3202 | ***TBX3* ^b^** |
| 6 | **rs9469089** | 32254635 | p21.32 | G/C | 0.1567 | 0.1237 | ***RNF5*** |
| 5 | rs32588 | 149180236 | q32 | T/C | 0.0548 | 1.0000 | ***PPARGC1B*** |
| 3 | rs4855881 | 49690450 | p21.31 | A/G | 0.0731 | 0.3840 | ***APEH*** |
| 18 | rs10515978 | 55156635 | q21.32 | A/G | 0.3123 | 0.2838 | ***LMAN1*** |
| 1 | rs601675 | 239250235 | q43 | A/G | 0.2641 | 0.4584 | ***RGS7*** |
| 7 | rs11761231 | 131020579 | q32.3 | A/G | 0.2492 | 0.5370 | ***PODXL* ^c^** |
| 14 | rs10129426 | 103088208 | q32.33 | C/T | 0.4219 | 1.0000 | ***BAG5* ^d^** |
| 2 | rs10201627 | 231482621 | q37.1 | G/T | 0.1063 | 0.7584 | ***GPR55*** |
| 16 | rs1049970 | 64989924 | q21 | C/T | 0.1900 | 0.3511 | ***CDH5*** |

a: Major/minor allele; b: rs1910047 is about 7 kb upstream of *TBX3;* c: rs11761231 is about 120kb upstream of *PODXL*; d: rs10129426 is about 4 kb downstream of *BAG5*.

**Table S4.** Three SNPs are in high LD with those in top-10 SNPs list.

| SNP | Position | Region | M/m | MAF | *P*_HWE_ | *P* ^a^ | Nearest  Gene | LD SNP ^b^ | *R^2^* |
| --- | --- | --- | --- | --- | --- | --- | --- | --- | --- |
| rs32586 | 149181113 | q32 | A/G | 0.0548 | 1.0000 | 1.11×10^-4^ | *PPARGC1B* | rs32588 | 1.00 |
| rs32589 | 149180082 | q32 | G/A | 0.0548 | 1.0000 | 1.11×10^-4^ | *PPARGC1B* | rs32588 | 1.00 |
| rs3749073 | 231483338 | q37.1 | C/A | 0.1080 | 0.7625 | 5.36×10^-4^ | *GPR55* | rs10201627 | 0.98 |

a: *P* value from multi-variable linear regression model including height, age and FEV_1_ at baseline, and average log transformed endotoxin level as covariates.

b: rs32588 and rs10201627 are in top-10 SNPs list.

**Table S5**. The association results of top-10 SNPs with or without first eight PCs adjustment.

| SNP | Without PCs adjustment | With PCs adjustment |
| --- | --- | --- |
|  | *P* ^a^ | *P* ^a^ |
| **rs1910047** | 3.70×10^-5^ | 1.47×10^-5^ |
| **rs9469089** | 6.19×10^-5^ | 7.71×10^-5^ |
| rs32588 | 1.11×10^-4^ | 6.54×10^-5^ |
| rs4855881 | 1.84×10^-4^ | 1.39×10^-4^ |
| rs10515978 | 3.16×10^-4^ | 4.01×10^-4^ |
| rs601675 | 3.32×10^-4^ | 5.83×10^-4^ |
| rs11761231 | 3.35×10^-4^ | 1.24×10^-4^ |
| rs10129426 | 3.88×10^-4^ | 2.58×10^-4^ |
| rs10201627 | 4.41×10^-4^ | 4.74×10^-4^ |
| rs1049970 | 4.63×10^-4^ | 7.87×10^-4^ |

a: Other covariates including height, age and FEV_1_ at baseline, and average log transformed endotoxin level.

**Table S6.** The association results of imputed SNPs and genotyped SNPs 500 kb around targeted SNPs. (Please download it from additional EXCEL file, **Supplementary File S2**)

**Table S7.** The results of association analysis using additive model.

| SNP | Genotype | *N* | Frequency(%) | Mean | ± | SD ^a^ | *β* (SE) ^b^ | *P* ^b^ |
| --- | --- | --- | --- | --- | --- | --- | --- | --- |
| **rs1910047** | TT | 248 | 82.39 | -4.32 | ± | 21.86 | -12.86 (3.27) | 1.04×10^-4^ |
|  | TA | 49 | 16.28 | -18.40 | ± | 35.81 |  |  |
|  | AA | 4 | 1.33 | -17.30 | ± | 15.38 |  |  |
| **rs9469089** | GG | 217 | 72.33 | -3.07 | ± | 23.35 | -9.45(2.58) | 2.90×10^-4^ |
|  | GC | 72 | 24.00 | -16.93 | ± | 28.51 |  |  |
|  | CC | 11 | 3.67 | -15.10 | ± | 13.98 |  |  |
| rs32588 | TT | 268 | 89.04 | -8.71 | ± | 23.60 | 17.21(4.39) | 1.11×10^-4^ |
|  | TC | 33 | 10.96 | 8.84 | ± | 31.18 |  |  |
|  | CC | 0 | 0.00 |  |  |  |  |  |
| rs4855881 | AA | 257 | 85.38 | -9.08 | ± | 22.28 | 14.83(3.92) | 1.84×10^-4^ |
|  | AG | 44 | 14.62 | 6.60 | ± | 34.96 |  |  |
|  | GG | 0 | 0.00 |  |  |  |  |  |
| rs10515978 | AA | 138 | 45.85 | -12.59 | ± | 23.71 | 5.34(2.20) | 1.59×10^-2^ |
|  | AG | 138 | 45.85 | -0.25 | ± | 25.18 |  |  |
|  | GG | 25 | 8.30 | -10.82 | ± | 24.18 |  |  |
| rs601675 | AA | 160 | 53.16 | -2.19 | ± | 18.74 | -7.20(2.32) | 2.07×10^-3^ |
|  | AG | 123 | 40.86 | -12.41 | ± | 31.59 |  |  |
|  | GG | 18 | 5.98 | -9.23 | ± | 15.24 |  |  |
| rs11761231 | AA | 167 | 55.48 | -11.54 | ± | 21.07 | 7.58(2.32) | 1.19×10^-3^ |
|  | AG | 118 | 39.20 | -0.71 | ± | 29.43 |  |  |
|  | GG | 16 | 5.32 | -1.98 | ± | 18.88 |  |  |
| rs10129426 | CC | 100 | 33.22 | -13.44 | ± | 27.26 | 6.43(1.99) | 1.34×10^-3^ |
|  | CT | 148 | 49.17 | -4.52 | ± | 24.30 |  |  |
|  | TT | 53 | 17.61 | -0.57 | ± | 20.22 |  |  |
| rs10201627 | GG | 241 | 80.07 | -4.23 | ± | 23.05 | -10.06(3.17) | 1.64×10^-3^ |
|  | GT | 56 | 18.60 | -18.32 | ± | 30.65 |  |  |
|  | TT | 4 | 1.33 | 0.81 | ± | 12.01 |  |  |
| rs1049970 | CC | 194 | 64.67 | -2.99 | ± | 23.16 | -8.67(2.59) | 9.23×10^-4^ |
|  | CT | 98 | 32.67 | -13.95 | ± | 27.43 |  |  |
|  | TT | 8 | 2.66 | -16.11 | ± | 17.88 |  |  |

a: Mean and standard deviation of the rate of FEV_1_ decline (ml/month); b: Coefficient, standard error and *P* value to SNP in linear regression model adjusted height, age and FEV_1_ at baseline, and average log transformed endotoxin level.

**Table S8.** The results of sensitivity analysis in those subjects with age ≥ 18 years.

| SNP | Subjects with age ≥ 18 years | | All subjects | |
| --- | --- | --- | --- | --- |
|  | *β* (SE) ^a^ | *P* ^a^ | *β* (SE) ^a^ | *P* ^a^ |
| **rs1910047** | -13.33(4.12) | 1.41×10^-3^ | -15.17(3.62) | 3.70×10^-5^ |
| **rs9469089** | -12.27(3.57) | 6.97×10^-4^ | -12.50(3.08) | 6.19×10^-5^ |
| rs32588 | 14.98(5.21) | 4.43×10^-3^ | 17.21(4.39) | 1.11×10^-4^ |
| rs4855881 | 17.19(4.70) | 3.22×10^-4^ | 14.83(3.92) | 1.84×10^-4^ |
| rs601675 | -9.02(3.28) | 6.50×10^-3^ | -10.14(2.79) | 3.32×10^-4^ |
| rs11761231 | 7.25(3.23) | 2.60×10^-2^ | 10.05(2.77) | 3.35×10^-4^ |
| rs10515978 | 8.78(3.21) | 6.74×10^-3^ | 10.09(2.77) | 3.16×10^-4^ |
| rs1049970 | -10.44(3.41) | 2.49×10^-2^ | -10.30(2.91) | 4.63×10^-4^ |
| rs10129426 | 9.56(3.42) | 5.69×10^-3^ | 10.52(2.93) | 3.88×10^-4^ |
| rs10201627 | -12.90(3.96) | 1.31×10^-3^ | -12.35(3.47) | 4.41×10^-4^ |

a: Coefficients, stand error and *P* value to SNP in linear regression model adjusted for height, age and FEV_1_ at baseline, and average log transformed endotoxin level.

**Table S9.** The results of genetic risk score analysis for top-10 SNPs.

| Genetic risk score | *N* | Mean | ± | SD ^a^ | *β* (SE) ^b^ | *P* ^b^ |
| --- | --- | --- | --- | --- | --- | --- |
| 0-2 | 32 | 21.63 | ± | 38.59 | Ref. |  |
| 3 | 39 | -1.78 | ± | 14.78 | -23.41(5.04) | 5.07×10^-6^ |
| 4 | 79 | -4.63 | ± | 14.96 | -24.44(4.46) | 9.02×10^-8^ |
| 5 | 66 | -8.77 | ± | 8.58 | -29.27(4.58) | 6.77×10^-10^ |
| 6 | 54 | -14.23 | ± | 18.27 | -34.91(4.73) | 1.71×10^-12^ |
| 7-10 | 31 | -30.72 | ± | 39.29 | -51.48(5.34) | 2.88×10^-19^ |
| **Trend test** |  |  |  |  |  | **3.01×10^-18^** |

a: Mean and stand deviation of the rate of FEV_1_ decline (ml/month); b: Coefficient, standard error and *P* value to genetic risk score in linear regression model adjusted for height, age and FEV_1_ at baseline, and average log transformed endotoxin level;

**Table S10.** Results of stratified genetic risk score analysis on top-10 SNPs.

| GRS ^a^ | Age < 18 years | | | | | | 18 ≤ Age < 25 years | | | | | | Age ≥ 25 years | | | | | |
| --- | --- | --- | --- | --- | --- | --- | --- | --- | --- | --- | --- | --- | --- | --- | --- | --- | --- | --- |
|  | *N* | Mean ± SD ^b^ | | | *β* (SE) ^c^ | *P* ^c^ | *N* | Mean ± SD ^b^ | | | *β* (SE) ^c^ | *P* | *N* | Mean ± SD ^b^ | | | *β* (SE) ^c^ | *P* ^c^ |
| 0-2 | 11 | 26.98 | ± | 28.58 | Ref. |  | 11 | 38.31 | ± | 47.07 | Ref. |  | 10 | -2.62 | ± | 27.06 | Ref. |  |
| 3 | 11 | 3.55 | ± | 19.36 | -23.58(8.25) | 5.74×10^-3^ | 16 | 0.01 | ± | 14.66 | -37.45(10.63) | 6.51×10^-4^ | 12 | -9.05 | ± | 5.46 | -7.04(5.71) | 2.20×10^-1^ |
| 4 | 16 | 0.02 | ± | 18.83 | -28.41(7.60) | 3.99×10^-4^ | 33 | -1.85 | ± | 17.14 | -32.92( 9.51) | 8.00×10^-4^ | 30 | -10.17 | ± | 6.48 | -7.74(4.86) | 1.14×10^-1^ |
| 5 | 15 | -3.35 | ± | 8.45 | -31.52(7.73) | 1.28×10^-4^ | 21 | -8.82 | ± | 6.94 | -44.20(10.08) | 2.90×10^-5^ | 30 | -11.46 | ± | 8.64 | -9.05(4.88) | 6.62×10^-2^ |
| 6 | 12 | -10.93 | ± | 17.88 | -38.31(8.08) | 1.22×10^-5^ | 16 | -19.46 | ± | 28.52 | -55.87(10.61) | 8.23×10^-7^ | 26 | -12.53 | ± | 7.29 | -11.12(4.98) | 2.77×10^-2^ |
| 7-10 | 8 | -26.21 | ± | 23.13 | -55.49 (9.09) | 6.80×10^-8^ | 10 | -37.91 | ± | 60.56 | -69.80(11.89) | 5.88×10^-8^ | 13 | -27.96 | ± | 27.06 | -24.93(5.67) | 2.52×10^-5^ |
| **Trend test** | |  |  |  |  | **4.69×10^-8^** |  |  |  |  |  | **2.74×10^-8^** |  |  |  |  |  | **9.61×10^-5^** |

a: Genetic risk score; b: Mean and stand deviation of the rate of FEV_1_ decline (ml/month); c: Coefficient, standard error and *P* value to GRS in linear regression model adjusted for height and FEV_1_ at baseline, and average log transformed endotoxin level.

**Table S10.** Results of stratified genetic risk score analysis on top-10 SNPs (Cont.).

| GRS ^a^ | Average endotoxin < 163 EU/m^3^ | | | | | | Average endotoxin ≥ 163 EU/m^3^ | | | | | |
| --- | --- | --- | --- | --- | --- | --- | --- | --- | --- | --- | --- | --- |
|  | *N* | Mean | ± | SD ^b^ | *β* (SE) ^c^ | *P* ^c^ | *N* | Mean | ± | SD ^b^ | *β* (SE) ^c^ | *P* ^c^ |
| 0-2 | 13 | 21.62 | ± | 51.10 | Ref. |  | 19 | 21.63 | ± | 28.71 | Ref. |  |
| 3 | 16 | -8.02 | ± | 5.73 | -28.74(8.61) | 1.08×10^-3^ | 23 | 2.56 | ± | 17.52 | -19.40(6.02) | 1.55×10^-3^ |
| 4 | 38 | -8.15 | ± | 10.62 | -26.78(7.47) | 4.64×10^-4^ | 41 | -1.37 | ± | 17.59 | -22.33(5.39) | 5.71×10^-5^ |
| 5 | 34 | -10.67 | ± | 8.57 | -32.07(7.67) | 5.12×10^-5^ | 32 | -6.76 | ± | 8.24 | -28.05(5.62) | 1.67×10^-6^ |
| 6 | 30 | -15.68 | ± | 21.94 | -37.63(7.68) | 2.63×10^-6^ | 24 | -12.41 | ± | 12.50 | -33.08(5.96) | 1.29×10^-7^ |
| 7-10 | 16 | -32.50 | ± | 44.51 | -53.49(8.78) | 1.03×10^-8^ | 15 | -28.81 | ± | 34.32 | -50.44(6.68) | 4.53×10^-12^ |
| **Trend test** |  |  |  |  |  | **5.29×10^-8^** |  |  |  |  |  | **2.52×10^-12^** |

a: Genetic risk score; b: Mean and stand deviation of the rate of FEV_1_ decline (ml/month); c: Coefficient, standard error and *P* value to GRS in linear regression model adjusted for age, height and FEV_1_ at baseline.

**Figure S1**. The FEV_1_ for each participant at different follow-up time.

1500

2000

2500

3000

3500

FEV_1_ (ml)

**0**

**3**

6

9

**12**

15

**18**

Follow-up time (Month)

**Figure S2**. Manhattan plot of *P* values in –log10 scale from dominant model on 27,611 SNPs using 301 individuals. The red line represents *P* = 1×10^-4^, and the blue line represents *P* = 5×10^-4^ above which the top-10 SNPs were selected.

**
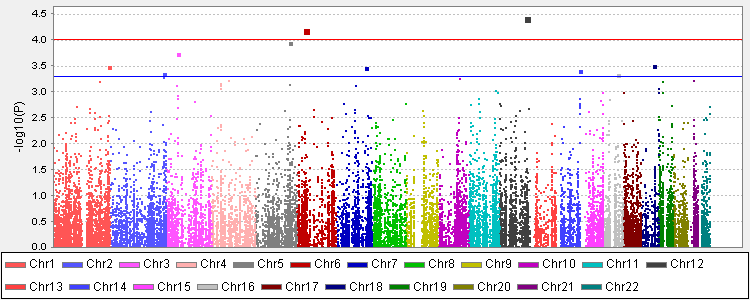
**

**Figure S3.** Quantile-Quantile plot was derived by *P* values in -log10 scale. *P* values were acquired from dominant model. Genomic control inflation factor (*λ*) is 1.026 in this study.

**
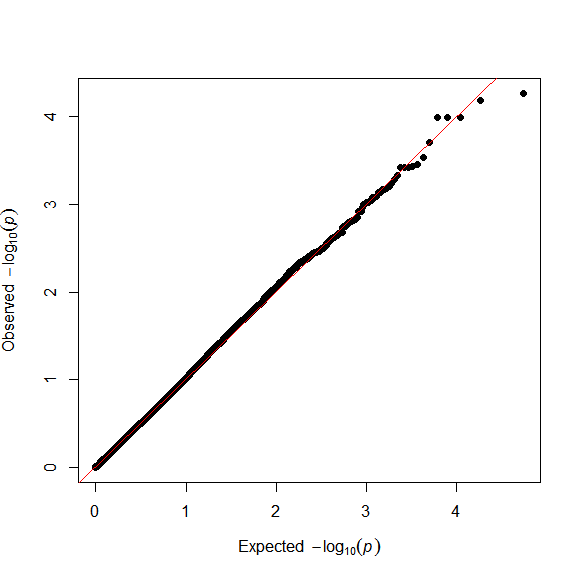
**
