# Supplementary material for: A Large Scale Gene-Centric Association Study of Lung Function in Newly-Hired Female Cotton Textile Workers with Endotoxin Exposure
Source: PLoS One. 2013 Mar 19;8(3):e59035. doi: 10.1371/journal.pone.0059035 (PMC3602449; doi:10.1371/journal.pone.0059035)
Supplement: Appendix S1 — Description of the FWER and FDR controlling procedure. (DOCX) [file pone.0059035.s003.docx]

**Appendix S1**

We adopted the methods described in Zhang X et al. (2010) [1] (page 219) to control false discovery rate (FDR) and family-wise error rate (FWER) in study-wide level. The FDR is the expected proportion of incorrectly rejected null hypotheses. While, the FWER is the overall type 1 error in a family of tests, which is also known as the probability of getting at least one false positive result. Thus, the FWER controlling procedure is thought to be more conservative. Here are the detailed steps of these two procedures in this study.

**The FDR Controlling Procedure**

(1) We randomly shuffled the outcome variable (the rate of FEV_1_ decline) for all participants, instead of SNPs to preserve the linkage disequilibrium (LD) structure among SNPs.

(2) For each permutation sample, we performed an association analysis via multi-variable linear regression model (MLRM) (see Equation 2 in the Statistical Methods section for details) to evaluate the significance of each SNP.

(3) We repeated the above steps for 5,000 times and pooled all association results from permutation samples together (total 27,611×5,000=1.38×10^8^ permutation statistics *Z*).

(4) The permutation *P* value for a SNP with statistic *z* in original sample is determined as the proportion of absolute permutation statistics *Z* no less than absolute *z*.

(5) The FDR q-value was determined using 27,611 permutation *P* values.

**The FWER Controlling Procedure**

The first two steps of FWER controlling procedure are the same as the FDR controlling procedure. The following steps are:

(3) The maximum statistic among all 27,611 SNPs was recorded.

(4) We repeated the above steps for 5,000 times to form the null distribution of the maximum statistic.

(5) Given an error rate threshold *α* (FWER), the critical value is the 100*α*-th% largest value in the null distribution.

(6) A SNP is considered significant if its statistic from MLRM exceeds the critical value in original sample.

**Reference**

1. Zhang X, Huang S, Zou F, Wang W (2010) TEAM: efficient two-locus epistasis tests in human genome-wide association study. Bioinformatics 26: i217-227.
