# Supplementary material for: A Large Scale Gene-Centric Association Study of Lung Function in Newly-Hired Female Cotton Textile Workers with Endotoxin Exposure
Source: PLoS One. 2013 Mar 19;8(3):e59035. doi: 10.1371/journal.pone.0059035 (PMC3602449; doi:10.1371/journal.pone.0059035)
Supplement: Appendix S2 — Regional plot of the top-10 SNP using P values derived from multi-variable linear regression model adjusted for height, age and FEV1 at baseline, and average log transformed endotoxin level. (PDF) [file pone.0059035.s004.pdf]

Appendix S2

Regional plot of the top-10 SNP using *P* values derived from multi-variable linear regression model adjusted for height, age and FEV_1 at baseline, and average log transformed endotoxin level were given below. Results (-log10 *P*) are shown for SNPs in the region flanking 500 kb on each side of targeted SNP. The LD - r^2 for each SNP were presented in different colors. The genes within the region of interest are annotated, with arrows indicating the direction of transcription.

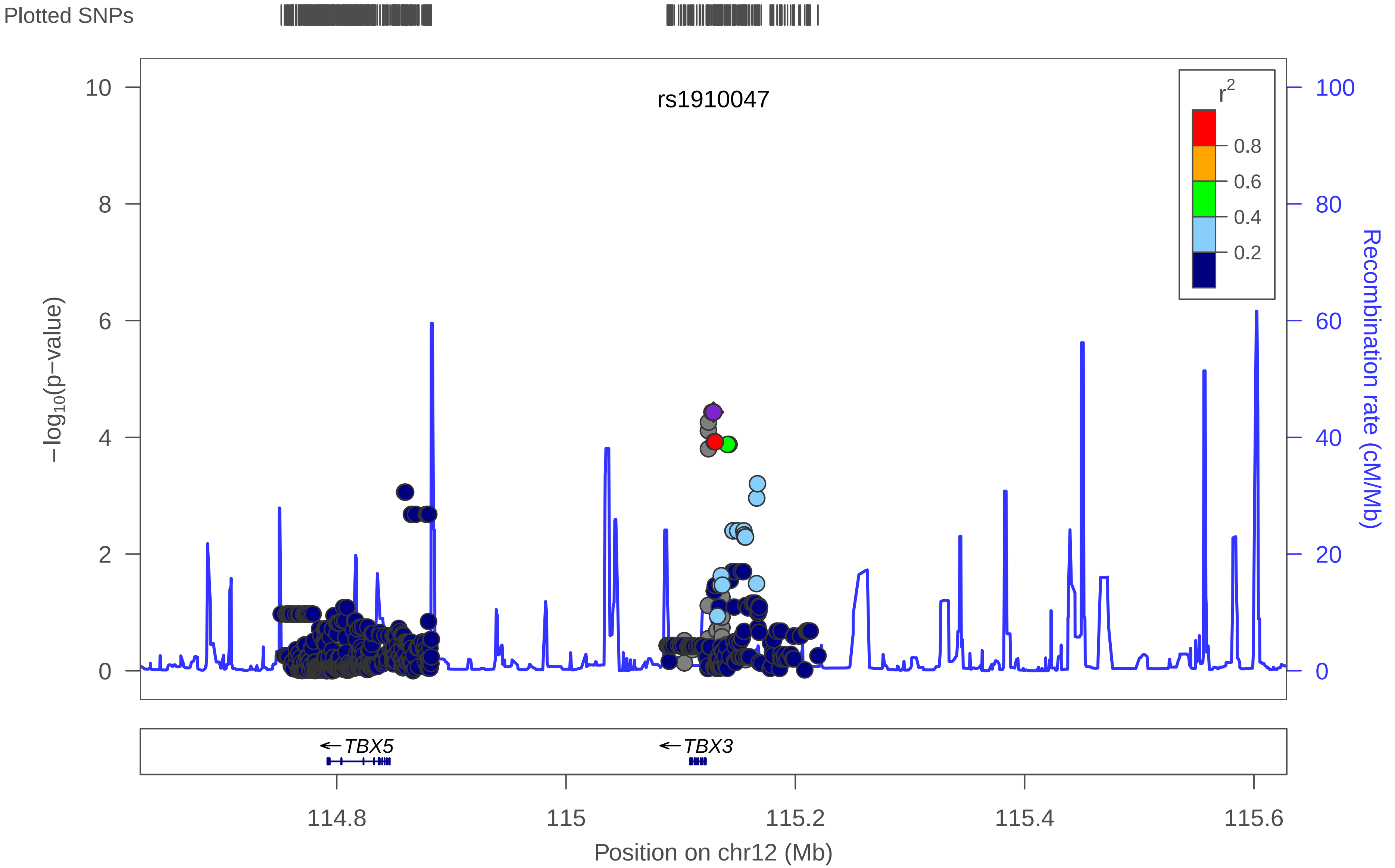

Plotted SNPs
rs1910047
r²
0.8
0.6
0.4
0.2
−log10(p−value)
Recombination rate (cM/Mb)
10
8
6
4
2
0
100
80
60
40
20
0
TBX5
TBX3
114.8
115
115.2
115.4
115.6
Position on chr12 (Mb)

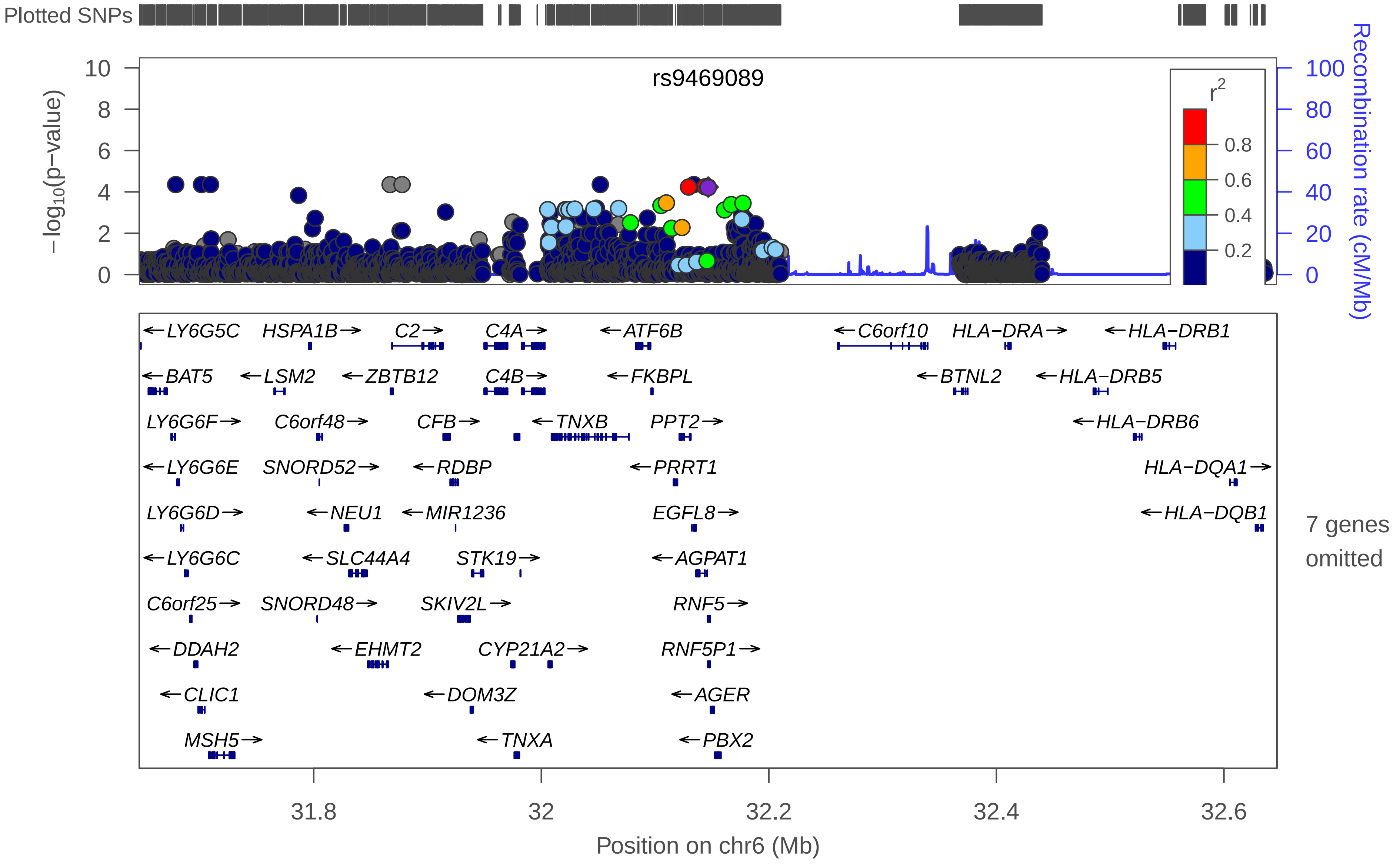
Plotted SNPs
rs9469089
−log10(p−value)
10
8
6
4
2
0
r2
0.8
0.6
0.4
0.2
100
80
60
40
20
0
Recombination rate (cM/Mb)
←LY6G5C
HSPA1B→
C2→
C4A→
←ATF6B
←C6orf10
HLA−DRA→
←HLA−DRB1
←BAT5
←LSM2
←ZBTB12
C4B→
←FKBPL
←BTNL2
←HLA−DRB5
LY6G6F→
C6orf48→
CFB→
←TNXB
PPT2→
←HLA−DRB6
←LY6G6E
SNORD52→
←RDBP
←PRRT1
HLA−DQA1→
LY6G6D→
←NEU1
←MIR1236
EGFL8→
←HLA−DQB1
←LY6G6C
←SLC44A4
STK19→
←AGPAT1
7 genes omitted
C6orf25→
SNORD48→
SKIV2L→
RNF5→
←DDAH2
←EHMT2
CYP21A2→
RNF5P1→
←CLIC1
←DOM3Z
←AGER
MSH5→
←TNXA
←PBX2
31.8
32
32.2
32.4
32.6
Position on chr6 (Mb)

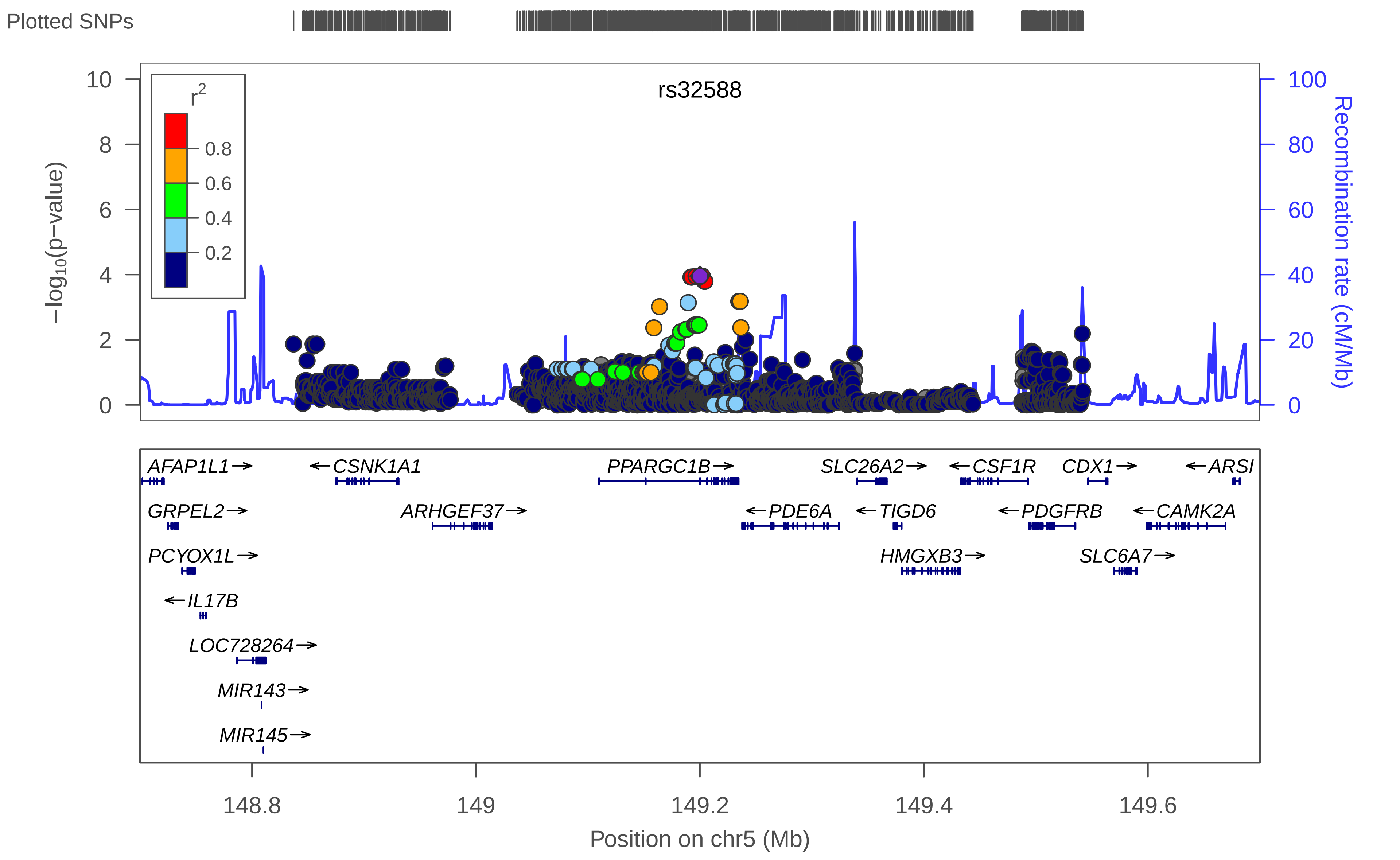

Plotted SNPs
rs32588
r2
0.8
0.6
0.4
0.2
−log10(p−value)
Recombination rate (cM/Mb)
AFAP1L1
CSNK1A1
PPARGC1B
SLC26A2
CSF1R
CDX1
ARSI
GRPEL2
ARHGEF37
PDE6A
TIGD6
PDGFRB
CAMK2A
PCYOX1L
HMGXB3
SLC6A7
IL17B
LOC728264
MIR143
MIR145
148.8
149
149.2
149.4
149.6
Position on chr5 (Mb)

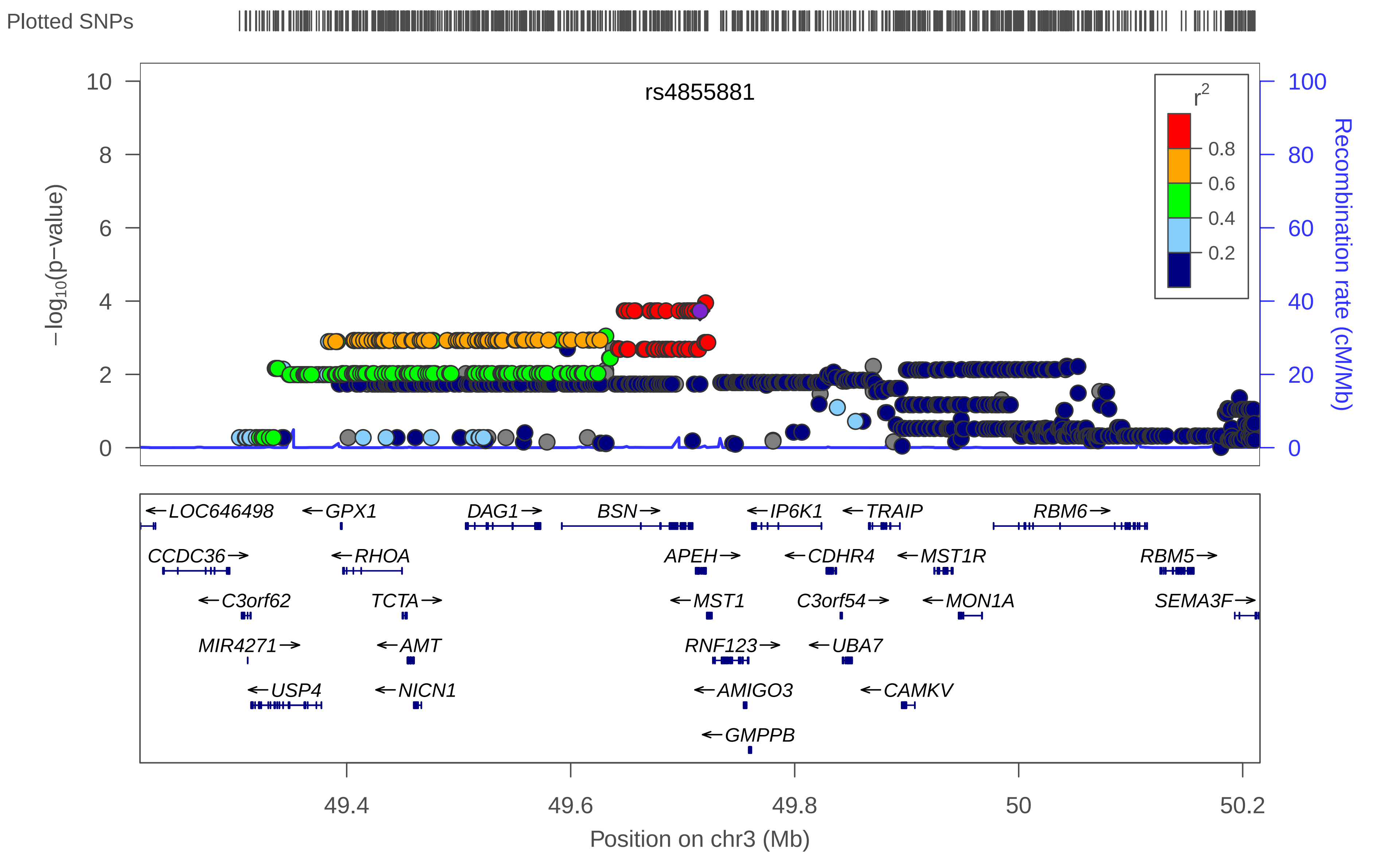

Plotted SNPs
rs4855881
r2
0.8
0.6
0.4
0.2
−log10(p−value)
Recombination rate (cM/Mb)
0
2
4
6
8
10
0
20
40
60
80
100
LOC646498
GPX1
DAG1
BSN
IP6K1
TRAIP
RBM6
CCDC36
RHOA
APEH
CDHR4
MST1R
RBM5
C3orf62
TCTA
MST1
C3orf54
MON1A
SEMA3F
MIR4271
AMT
RNF123
UBA7
USP4
NICN1
AMIGO3
CAMKV
GMPPB
49.4
49.6
49.8
50
50.2
Position on chr3 (Mb)

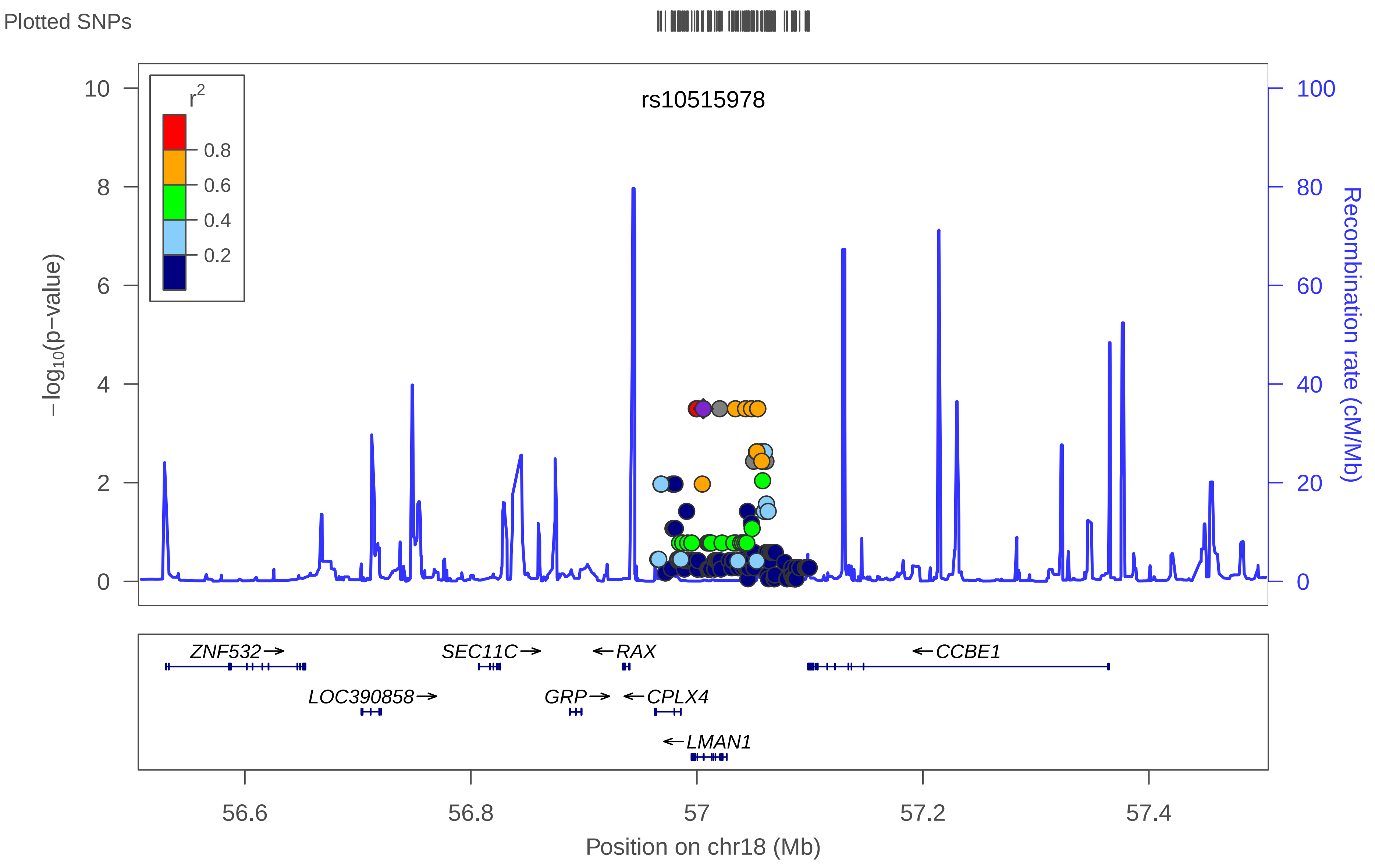

Plotted SNPs
rs10515978
r2
0.8
0.6
0.4
0.2
10
8
6
4
2
0
−log10(p−value)
100
80
60
40
20
0
Recombination rate (cM/Mb)
ZNF532
SEC11C
RAX
CCBE1
LOC390858
GRP
CPLX4
LMAN1
56.6
56.8
57
57.2
57.4
Position on chr18 (Mb)

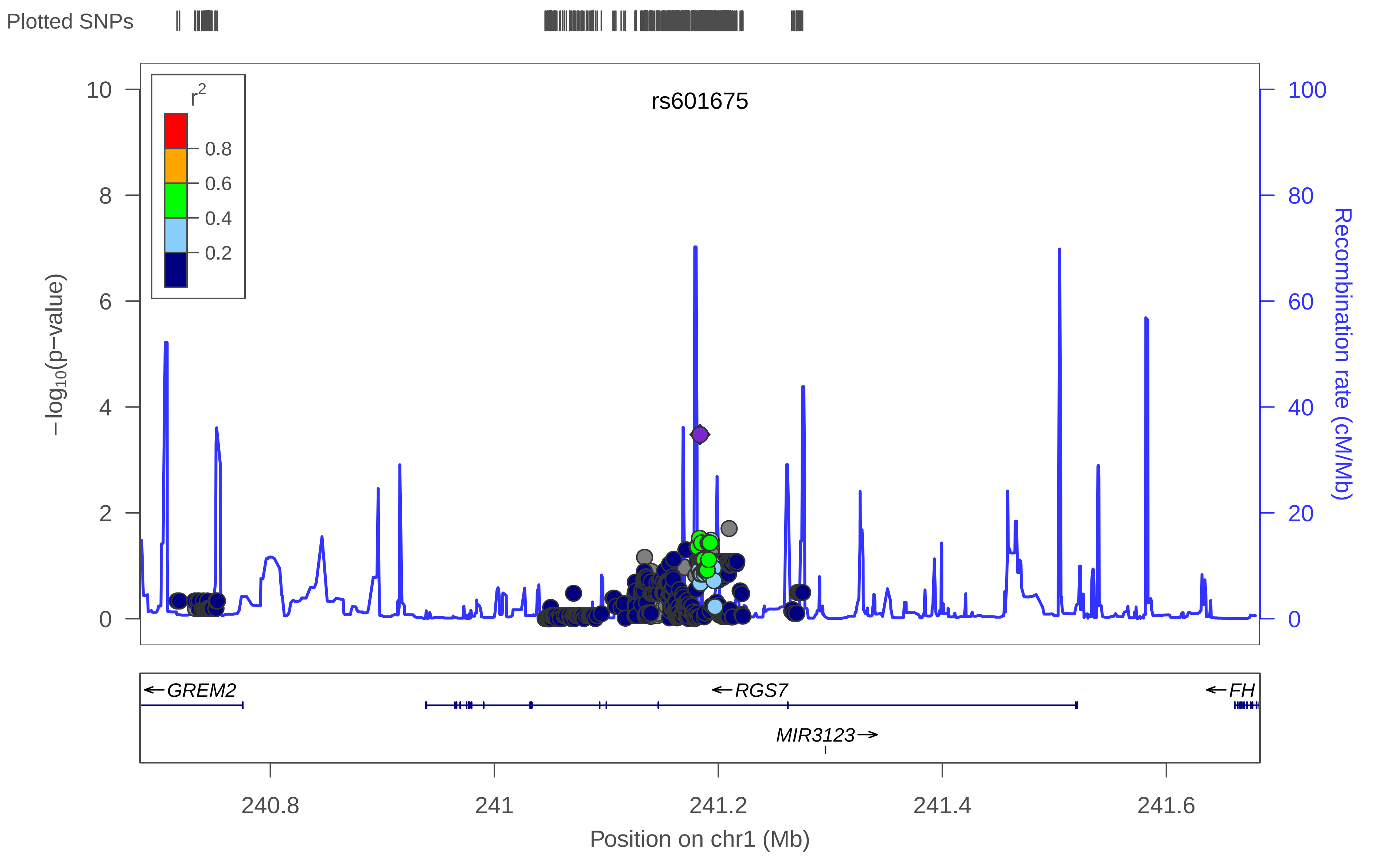

Plotted SNPs
rs601675
r2
0.8
0.6
0.4
0.2
−log10(p−value)
Recombination rate (cM/Mb)
10
8
6
4
2
0
100
80
60
40
20
0
←GREM2
←RGS7
←FH
MIR3123→
240.8
241
241.2
241.4
241.6
Position on chr1 (Mb)

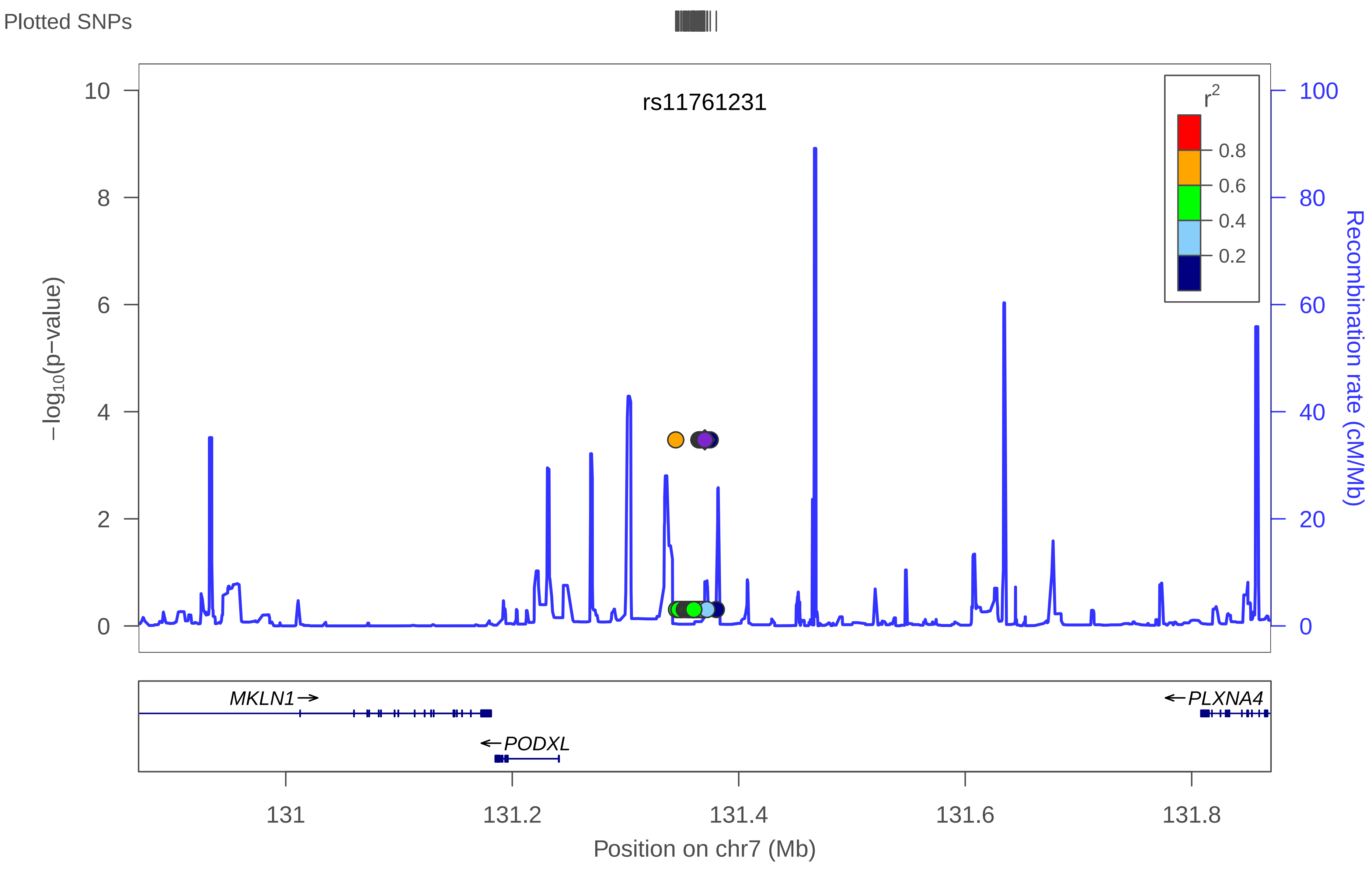

Plotted SNPs
rs11761231
r2
0.8
0.6
0.4
0.2
10
8
6
4
2
0
100
80
60
40
20
0
−log10(p−value)
Recombination rate (cM/Mb)
MKLN1→
←PODXL
←PLXNA4
131
131.2
131.4
131.6
131.8
Position on chr7 (Mb)

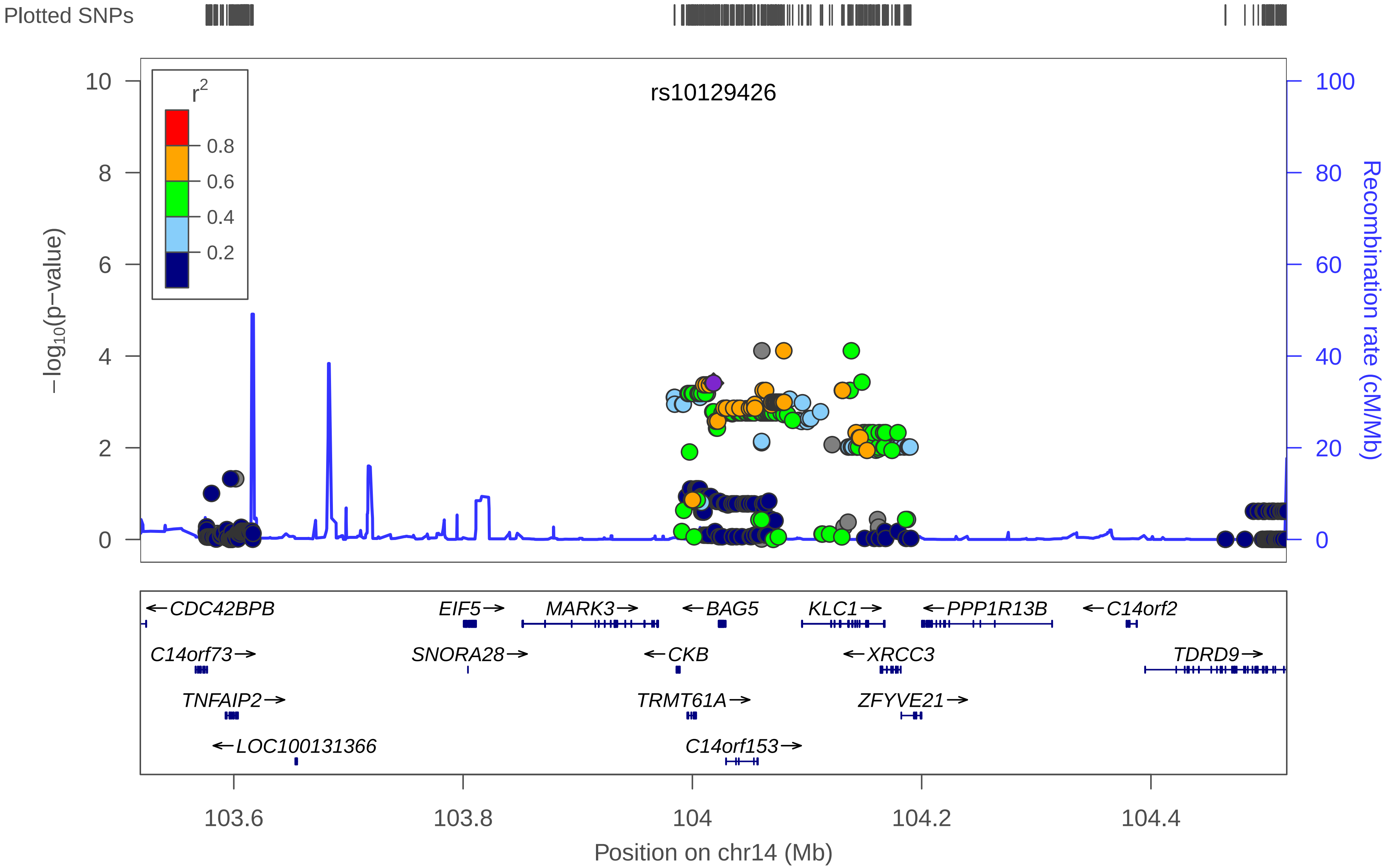

Plotted SNPs
rs10129426
r2
0.8
0.6
0.4
0.2
10
8
6
4
2
0
−log10(p−value)
100
80
60
40
20
0
Recombination rate (cM/Mb)
←CDC42BPB
EIF5→
MARK3→
←BAG5
KLC1→
←PPP1R13B
←C14orf2
C14orf73→
SNORA28→
←CKB
←XRCC3
TDRD9→
TNFAIP2→
TRMT61A→
ZFYVE21→
←LOC100131366
C14orf153→
103.6
103.8
104
104.2
104.4
Position on chr14 (Mb)

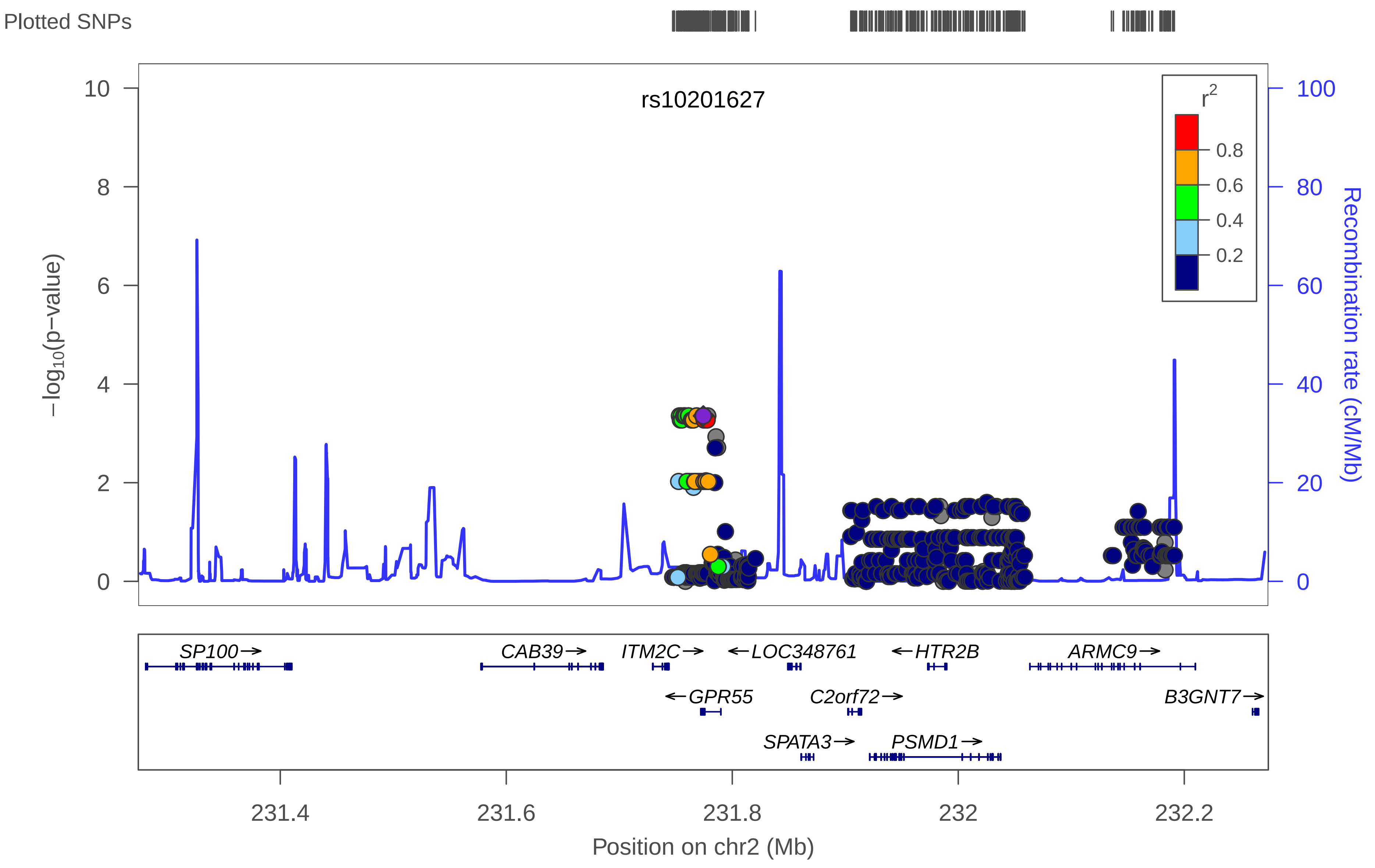

Plotted SNPs
rs10201627
r2
0.8
0.6
0.4
0.2
−log10(p−value)
Recombination rate (cM/Mb)
0
2
4
6
8
10
20
40
60
80
100
SP100→
CAB39→
ITM2C→
←LOC348761
←HTR2B
ARMC9→
←GPR55
C2orf72→
B3GNT7→
SPATA3→
PSMD1→
231.4
231.6
231.8
232
232.2
Position on chr2 (Mb)

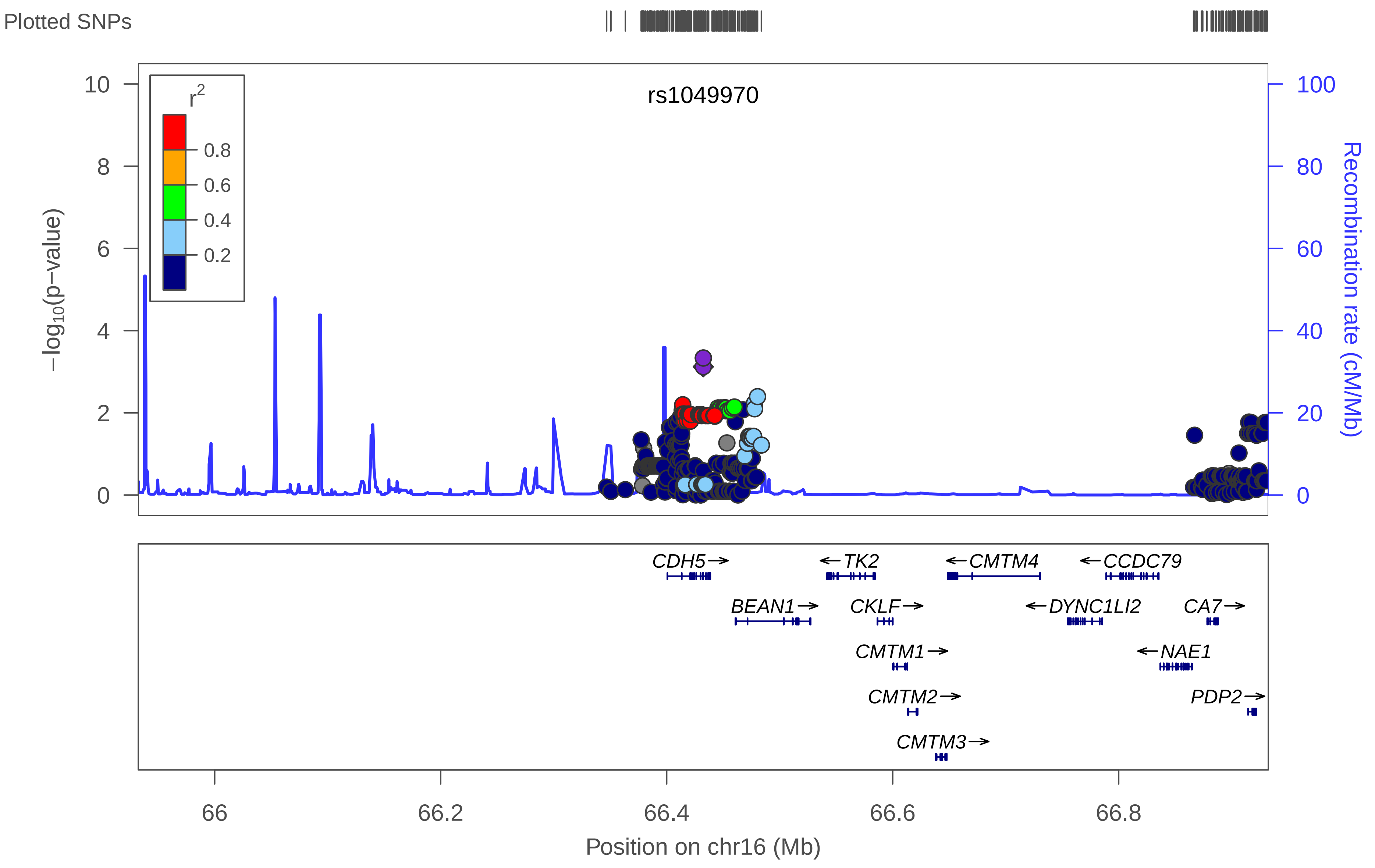

Plotted SNPs
rs1049970
r2
0.8
0.6
0.4
0.2
−log10(p−value)
Recombination rate (cM/Mb)
CDH5
TK2
CMTM4
CCDC79
BEAN1
CKLF
DYNC1LI2
CA7
CMTM1
NAE1
CMTM2
PDP2
CMTM3
66
66.2
66.4
66.6
66.8
Position on chr16 (Mb)
