# Supplementary material for: A Large Scale Gene-Centric Association Study of Lung Function in Newly-Hired Female Cotton Textile Workers with Endotoxin Exposure
Source: PLoS One. 2013 Mar 19;8(3):e59035. doi: 10.1371/journal.pone.0059035 (PMC3602449; doi:10.1371/journal.pone.0059035)
Supplement: Appendix S3 — Sixty-three nodes in the gene-gene interaction functional network. (DOCX) [file pone.0059035.s005.docx]

**Appendix S3**

There are 63 nodes in the gene-gene interaction functional network. The table below gives symbol, name, localization and protein function of each node. The nodes are in alphabetical order.

| **ID** | **Symbol** | [**Name**](javascript:%20void(0)) | [**Localizations**](javascript:%20void(0)) | **Protein function** |
| --- | --- | --- | --- | --- |
| 1 | [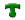](https://portal.genego.com/cgi/regulation/regulation_info.cgi?id=835) | [Adrenomedullin](https://portal.genego.com/cgi/regulation/regulation_info.cgi?id=835) | [extracellular region](https://portal.genego.com/cgi/view_tree.cgi?type=loc&id=23&channel=Other&section=Network%20Statistics) | Receptor ligand |
| 2 | [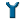](https://portal.genego.com/cgi/regulation/regulation_info.cgi?id=6164) | [ALK-2](https://portal.genego.com/cgi/regulation/regulation_info.cgi?id=6164) | [membrane](https://portal.genego.com/cgi/view_tree.cgi?type=loc&id=8&channel=Other&section=Network%20Statistics) | Generic receptor |
| 3 | [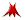](https://portal.genego.com/cgi/regulation/regulation_info.cgi?id=35) | [AP-1](https://portal.genego.com/cgi/regulation/regulation_info.cgi?id=35) | [nucleus](https://portal.genego.com/cgi/view_tree.cgi?type=loc&id=43&channel=Other&section=Network%20Statistics) | Transcription factor |
| 4 | [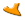](https://portal.genego.com/cgi/regulation/regulation_info.cgi?id=-2142100420) | [APEH](https://portal.genego.com/cgi/regulation/regulation_info.cgi?id=-2142100420) | [cytoplasm](https://portal.genego.com/cgi/view_tree.cgi?type=loc&id=1&channel=Other&section=Network%20Statistics) | Genetic protease |
| 5 | [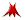](https://portal.genego.com/cgi/regulation/regulation_info.cgi?id=-753149152) | [ATF-6 alpha](https://portal.genego.com/cgi/regulation/regulation_info.cgi?id=-753149152) | [nucleus](https://portal.genego.com/cgi/view_tree.cgi?type=loc&id=43&channel=Other&section=Network%20Statistics) | Transcription factor |
| 6 | [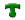](https://portal.genego.com/cgi/regulation/regulation_info.cgi?id=6102) | [BMP2](https://portal.genego.com/cgi/regulation/regulation_info.cgi?id=6102) | [extracellular region](https://portal.genego.com/cgi/view_tree.cgi?type=loc&id=23&channel=Other&section=Network%20Statistics) | Receptor ligand |
| 7 | [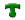](https://portal.genego.com/cgi/regulation/regulation_info.cgi?id=6108) | [BMP4](https://portal.genego.com/cgi/regulation/regulation_info.cgi?id=6108) | [extracellular region](https://portal.genego.com/cgi/view_tree.cgi?type=loc&id=23&channel=Other&section=Network%20Statistics) | Receptor ligand |
| 8 | [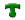](https://portal.genego.com/cgi/regulation/regulation_info.cgi?id=7038) | [BMP7](https://portal.genego.com/cgi/regulation/regulation_info.cgi?id=7038) | [extracellular region](https://portal.genego.com/cgi/view_tree.cgi?type=loc&id=23&channel=Other&section=Network%20Statistics) | Receptor ligand |
| 9 | [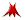](https://portal.genego.com/cgi/regulation/regulation_info.cgi?id=2257) | [c-Myc](https://portal.genego.com/cgi/regulation/regulation_info.cgi?id=2257) | [nucleus](https://portal.genego.com/cgi/view_tree.cgi?type=loc&id=43&channel=Other&section=Network%20Statistics) | Transcription factor |
| 10 | [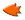](https://portal.genego.com/cgi/regulation/regulation_info.cgi?id=887) | [c-Src](https://portal.genego.com/cgi/regulation/regulation_info.cgi?id=887) | [cytoplasm](https://portal.genego.com/cgi/view_tree.cgi?type=loc&id=1&channel=Other&section=Network%20Statistics) | Protein kinase |
| 11 | [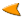](https://portal.genego.com/cgi/regulation/regulation_info.cgi?id=112) | [CBP](https://portal.genego.com/cgi/regulation/regulation_info.cgi?id=112) | [nucleus](https://portal.genego.com/cgi/view_tree.cgi?type=loc&id=43&channel=Other&section=Network%20Statistics) | Genetic enzyme |
| 12 | [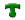](https://portal.genego.com/cgi/regulation/regulation_info.cgi?id=232) | [EGF](https://portal.genego.com/cgi/regulation/regulation_info.cgi?id=232) | [extracellular region](https://portal.genego.com/cgi/view_tree.cgi?type=loc&id=23&channel=Other&section=Network%20Statistics) | Receptor ligand |
| 13 | [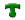](https://portal.genego.com/cgi/regulation/regulation_info.cgi?id=4507) | [Endothelin-1](https://portal.genego.com/cgi/regulation/regulation_info.cgi?id=4507) | [extracellular region](https://portal.genego.com/cgi/view_tree.cgi?type=loc&id=23&channel=Other&section=Network%20Statistics) | Receptor ligand |
| 14 | [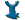](https://portal.genego.com/cgi/regulation/regulation_info.cgi?id=247) | [ErbB2](https://portal.genego.com/cgi/regulation/regulation_info.cgi?id=247) | [membrane](https://portal.genego.com/cgi/view_tree.cgi?type=loc&id=8&channel=Other&section=Network%20Statistics) | Receptor with enzyme |
| 15 | [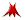](https://portal.genego.com/cgi/regulation/regulation_info.cgi?id=9097) | [ESR1 (nuclear)](https://portal.genego.com/cgi/regulation/regulation_info.cgi?id=9097) | [nucleus](https://portal.genego.com/cgi/view_tree.cgi?type=loc&id=43&channel=Other&section=Network%20Statistics) | Transcription factor |
| 16 | [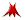](https://portal.genego.com/cgi/regulation/regulation_info.cgi?id=-1193216337) | [ETS](https://portal.genego.com/cgi/regulation/regulation_info.cgi?id=-1193216337) | [nucleus](https://portal.genego.com/cgi/view_tree.cgi?type=loc&id=43&channel=Other&section=Network%20Statistics) | Transcription factor |
| 17 | [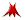](https://portal.genego.com/cgi/regulation/regulation_info.cgi?id=6351) | [FOXC2](https://portal.genego.com/cgi/regulation/regulation_info.cgi?id=6351) | [nucleus](https://portal.genego.com/cgi/view_tree.cgi?type=loc&id=43&channel=Other&section=Network%20Statistics) | Transcription factor |
| 18 | [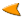](https://portal.genego.com/cgi/regulation/regulation_info.cgi?id=2539) | [HDAC3](https://portal.genego.com/cgi/regulation/regulation_info.cgi?id=2539) | [nucleus](https://portal.genego.com/cgi/view_tree.cgi?type=loc&id=43&channel=Other&section=Network%20Statistics) | Genetic enzyme |
| 19 | [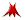](https://portal.genego.com/cgi/regulation/regulation_info.cgi?id=-1998745163) | [HNF3](https://portal.genego.com/cgi/regulation/regulation_info.cgi?id=-1998745163) | [nucleus](https://portal.genego.com/cgi/view_tree.cgi?type=loc&id=43&channel=Other&section=Network%20Statistics) | Transcription factor |
| 20 | [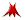](https://portal.genego.com/cgi/regulation/regulation_info.cgi?id=6018) | [HNF4-alpha](https://portal.genego.com/cgi/regulation/regulation_info.cgi?id=6018) | [nucleus](https://portal.genego.com/cgi/view_tree.cgi?type=loc&id=43&channel=Other&section=Network%20Statistics) | Transcription factor |
| 21 | [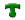](https://portal.genego.com/cgi/regulation/regulation_info.cgi?id=414) | [IL-8](https://portal.genego.com/cgi/regulation/regulation_info.cgi?id=414) | [extracellular region](https://portal.genego.com/cgi/view_tree.cgi?type=loc&id=23&channel=Other&section=Network%20Statistics) | Receptor ligand |
| 22 | [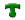](https://portal.genego.com/cgi/regulation/regulation_info.cgi?id=4096) | [Jagged1](https://portal.genego.com/cgi/regulation/regulation_info.cgi?id=4096) | [membrane](https://portal.genego.com/cgi/view_tree.cgi?type=loc&id=8&channel=Other&section=Network%20Statistics) | Receptor ligand |
| 23 | [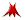](https://portal.genego.com/cgi/regulation/regulation_info.cgi?id=-2007163176) | [KLF4](https://portal.genego.com/cgi/regulation/regulation_info.cgi?id=-2007163176) | [nucleus](https://portal.genego.com/cgi/view_tree.cgi?type=loc&id=43&channel=Other&section=Network%20Statistics) | Transcription factor |
| 24 | [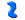](https://portal.genego.com/cgi/regulation/regulation_info.cgi?id=-1623930644) | [LMAN1](https://portal.genego.com/cgi/regulation/regulation_info.cgi?id=-1623930644) | [cytoplasm](https://portal.genego.com/cgi/view_tree.cgi?type=loc&id=1&channel=Other&section=Network%20Statistics) | Genetic binding protein |
| 25 | [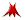](https://portal.genego.com/cgi/regulation/regulation_info.cgi?id=522) | [NF-kB](https://portal.genego.com/cgi/regulation/regulation_info.cgi?id=522) | [nucleus](https://portal.genego.com/cgi/view_tree.cgi?type=loc&id=43&channel=Other&section=Network%20Statistics) | Transcription factor |
| 26 | [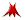](https://portal.genego.com/cgi/regulation/regulation_info.cgi?id=4336) | [NF-Y](https://portal.genego.com/cgi/regulation/regulation_info.cgi?id=4336) | [nucleus](https://portal.genego.com/cgi/view_tree.cgi?type=loc&id=43&channel=Other&section=Network%20Statistics) | Transcription factor |
| 27 | [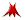](https://portal.genego.com/cgi/regulation/regulation_info.cgi?id=6409) | [NRL](https://portal.genego.com/cgi/regulation/regulation_info.cgi?id=6409) | [nucleus](https://portal.genego.com/cgi/view_tree.cgi?type=loc&id=43&channel=Other&section=Network%20Statistics) | Transcription factor |
| 28 | [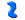](https://portal.genego.com/cgi/regulation/regulation_info.cgi?id=-1816309549) | [Nucleolin](https://portal.genego.com/cgi/regulation/regulation_info.cgi?id=-1816309549) | [nucleus](https://portal.genego.com/cgi/view_tree.cgi?type=loc&id=43&channel=Other&section=Network%20Statistics) | Genetic binding protein |
| 29 | [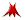](https://portal.genego.com/cgi/regulation/regulation_info.cgi?id=-115825747) | [Oct-3/4](https://portal.genego.com/cgi/regulation/regulation_info.cgi?id=-115825747) | [nucleus](https://portal.genego.com/cgi/view_tree.cgi?type=loc&id=43&channel=Other&section=Network%20Statistics) | Transcription factor |
| 30 | [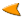](https://portal.genego.com/cgi/regulation/regulation_info.cgi?id=2201) | [p300](https://portal.genego.com/cgi/regulation/regulation_info.cgi?id=2201) | [nucleus](https://portal.genego.com/cgi/view_tree.cgi?type=loc&id=43&channel=Other&section=Network%20Statistics) | Genetic enzyme |
| 31 | [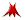](https://portal.genego.com/cgi/regulation/regulation_info.cgi?id=1075) | [p53](https://portal.genego.com/cgi/regulation/regulation_info.cgi?id=1075) | [nucleus](https://portal.genego.com/cgi/view_tree.cgi?type=loc&id=43&channel=Other&section=Network%20Statistics) | Transcription factor |
| 32 | [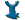](https://portal.genego.com/cgi/regulation/regulation_info.cgi?id=572) | [PDGF receptor](https://portal.genego.com/cgi/regulation/regulation_info.cgi?id=572) | [membrane](https://portal.genego.com/cgi/view_tree.cgi?type=loc&id=8&channel=Other&section=Network%20Statistics) | Receptor with enzyme |
| 33 | [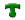](https://portal.genego.com/cgi/regulation/regulation_info.cgi?id=571) | [PDGF-A](https://portal.genego.com/cgi/regulation/regulation_info.cgi?id=571) | [extracellular region](https://portal.genego.com/cgi/view_tree.cgi?type=loc&id=23&channel=Other&section=Network%20Statistics) | Receptor ligand |
| 34 | [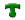](https://portal.genego.com/cgi/regulation/regulation_info.cgi?id=4560) | [PDGF-B](https://portal.genego.com/cgi/regulation/regulation_info.cgi?id=4560) | [extracellular region](https://portal.genego.com/cgi/view_tree.cgi?type=loc&id=23&channel=Other&section=Network%20Statistics) | Receptor ligand |
| 35 | [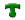](https://portal.genego.com/cgi/regulation/regulation_info.cgi?id=-170444462) | [PDGF-D](https://portal.genego.com/cgi/regulation/regulation_info.cgi?id=-170444462) | [extracellular region](https://portal.genego.com/cgi/view_tree.cgi?type=loc&id=23&channel=Other&section=Network%20Statistics) | Receptor ligand |
| 36 | [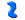](https://portal.genego.com/cgi/regulation/regulation_info.cgi?id=2644) | [PECAM1](https://portal.genego.com/cgi/regulation/regulation_info.cgi?id=2644) | [membrane](https://portal.genego.com/cgi/view_tree.cgi?type=loc&id=8&channel=Other&section=Network%20Statistics) | Genetic binding protein |
| 37 | [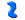](https://portal.genego.com/cgi/regulation/regulation_info.cgi?id=-1514121656) | [PERC](https://portal.genego.com/cgi/regulation/regulation_info.cgi?id=-1514121656) | [nucleus](https://portal.genego.com/cgi/view_tree.cgi?type=loc&id=43&channel=Other&section=Network%20Statistics) | Genetic binding protein |
| 38 | [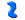](https://portal.genego.com/cgi/regulation/regulation_info.cgi?id=-1282116881) | [Podocalyxin-like 1](https://portal.genego.com/cgi/regulation/regulation_info.cgi?id=-1282116881) | [membrane](https://portal.genego.com/cgi/view_tree.cgi?type=loc&id=8&channel=Other&section=Network%20Statistics) | Genetic binding protein |
| 39 | [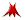](https://portal.genego.com/cgi/regulation/regulation_info.cgi?id=-1483525193) | [PPAR-gamma/RXR-alpha](https://portal.genego.com/cgi/regulation/regulation_info.cgi?id=-1483525193) | [nucleus](https://portal.genego.com/cgi/view_tree.cgi?type=loc&id=43&channel=Other&section=Network%20Statistics) | Transcription factor |
| 40 | [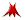](https://portal.genego.com/cgi/regulation/regulation_info.cgi?id=1100) | [Progesterone receptor](https://portal.genego.com/cgi/regulation/regulation_info.cgi?id=1100) | [nucleus](https://portal.genego.com/cgi/view_tree.cgi?type=loc&id=43&channel=Other&section=Network%20Statistics) | Transcription factor |
| 41 | [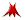](https://portal.genego.com/cgi/regulation/regulation_info.cgi?id=-461623326) | [PROX1](https://portal.genego.com/cgi/regulation/regulation_info.cgi?id=-461623326) | [nucleus](https://portal.genego.com/cgi/view_tree.cgi?type=loc&id=43&channel=Other&section=Network%20Statistics) | Transcription factor |
| 42 | [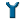](https://portal.genego.com/cgi/regulation/regulation_info.cgi?id=-1657739851) | [PTPR-mu](https://portal.genego.com/cgi/regulation/regulation_info.cgi?id=-1657739851) | [membrane](https://portal.genego.com/cgi/view_tree.cgi?type=loc&id=8&channel=Other&section=Network%20Statistics) | Generic receptor |
| 43 | [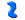](https://portal.genego.com/cgi/regulation/regulation_info.cgi?id=2093) | [Shc](https://portal.genego.com/cgi/regulation/regulation_info.cgi?id=2093) | [cytoplasm](https://portal.genego.com/cgi/view_tree.cgi?type=loc&id=1&channel=Other&section=Network%20Statistics) | Genetic binding protein |
| 44 | [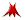](https://portal.genego.com/cgi/regulation/regulation_info.cgi?id=-1659642710) | [SLUG](https://portal.genego.com/cgi/regulation/regulation_info.cgi?id=-1659642710) | [nucleus](https://portal.genego.com/cgi/view_tree.cgi?type=loc&id=43&channel=Other&section=Network%20Statistics) | Transcription factor |
| 45 | [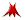](https://portal.genego.com/cgi/regulation/regulation_info.cgi?id=6436) | [SMAD1](https://portal.genego.com/cgi/regulation/regulation_info.cgi?id=6436) | [nucleus](https://portal.genego.com/cgi/view_tree.cgi?type=loc&id=43&channel=Other&section=Network%20Statistics) | Transcription factor |
| 46 | [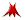](https://portal.genego.com/cgi/regulation/regulation_info.cgi?id=4322) | [SMAD3](https://portal.genego.com/cgi/regulation/regulation_info.cgi?id=4322) | [nucleus](https://portal.genego.com/cgi/view_tree.cgi?type=loc&id=43&channel=Other&section=Network%20Statistics) | Transcription factor |
| 47 | [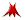](https://portal.genego.com/cgi/regulation/regulation_info.cgi?id=-50872036) | [SNAIL1](https://portal.genego.com/cgi/regulation/regulation_info.cgi?id=-50872036) | [nucleus](https://portal.genego.com/cgi/view_tree.cgi?type=loc&id=43&channel=Other&section=Network%20Statistics) | Transcription factor |
| 48 | [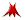](https://portal.genego.com/cgi/regulation/regulation_info.cgi?id=4297) | [SOX9](https://portal.genego.com/cgi/regulation/regulation_info.cgi?id=4297) | [nucleus](https://portal.genego.com/cgi/view_tree.cgi?type=loc&id=43&channel=Other&section=Network%20Statistics) | Transcription factor |
| 49 | [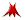](https://portal.genego.com/cgi/regulation/regulation_info.cgi?id=2498) | [SP1](https://portal.genego.com/cgi/regulation/regulation_info.cgi?id=2498) | [nucleus](https://portal.genego.com/cgi/view_tree.cgi?type=loc&id=43&channel=Other&section=Network%20Statistics) | Transcription factor |
| 50 | [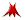](https://portal.genego.com/cgi/regulation/regulation_info.cgi?id=2554) | [SP3](https://portal.genego.com/cgi/regulation/regulation_info.cgi?id=2554) | [nucleus](https://portal.genego.com/cgi/view_tree.cgi?type=loc&id=43&channel=Other&section=Network%20Statistics) | Transcription factor |
| 51 | [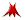](https://portal.genego.com/cgi/regulation/regulation_info.cgi?id=722) | [STAT3](https://portal.genego.com/cgi/regulation/regulation_info.cgi?id=722) | [nucleus](https://portal.genego.com/cgi/view_tree.cgi?type=loc&id=43&channel=Other&section=Network%20Statistics) | Transcription factor |
| 52 | [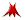](https://portal.genego.com/cgi/regulation/regulation_info.cgi?id=4267) | [STAT6](https://portal.genego.com/cgi/regulation/regulation_info.cgi?id=4267) | [nucleus](https://portal.genego.com/cgi/view_tree.cgi?type=loc&id=43&channel=Other&section=Network%20Statistics) | Transcription factor |
| 53 | [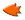](https://portal.genego.com/cgi/regulation/regulation_info.cgi?id=742) | [Syk](https://portal.genego.com/cgi/regulation/regulation_info.cgi?id=742) | [cytoplasm](https://portal.genego.com/cgi/view_tree.cgi?type=loc&id=1&channel=Other&section=Network%20Statistics) | Protein kinase |
| 54 | [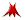](https://portal.genego.com/cgi/regulation/regulation_info.cgi?id=-1882774929) | [T3Rbeta/RXR-alpha](https://portal.genego.com/cgi/regulation/regulation_info.cgi?id=-1882774929) | [nucleus](https://portal.genego.com/cgi/view_tree.cgi?type=loc&id=43&channel=Other&section=Network%20Statistics) | Transcription factor |
| 55 | [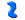](https://portal.genego.com/cgi/regulation/regulation_info.cgi?id=4608) | [TAL1](https://portal.genego.com/cgi/regulation/regulation_info.cgi?id=4608) | [cytoplasm](https://portal.genego.com/cgi/view_tree.cgi?type=loc&id=1&channel=Other&section=Network%20Statistics) | Genetic binding protein |
| 56 | [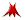](https://portal.genego.com/cgi/regulation/regulation_info.cgi?id=-1695582466) | [TBX3](https://portal.genego.com/cgi/regulation/regulation_info.cgi?id=-1695582466) | [nucleus](https://portal.genego.com/cgi/view_tree.cgi?type=loc&id=43&channel=Other&section=Network%20Statistics) | Transcription factor |
| 57 | [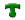](https://portal.genego.com/cgi/regulation/regulation_info.cgi?id=756) | [TGF-beta 1](https://portal.genego.com/cgi/regulation/regulation_info.cgi?id=756) | [extracellular region](https://portal.genego.com/cgi/view_tree.cgi?type=loc&id=23&channel=Other&section=Network%20Statistics) | Receptor ligand |
| 58 | [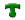](https://portal.genego.com/cgi/regulation/regulation_info.cgi?id=4567) | [TGF-beta 2](https://portal.genego.com/cgi/regulation/regulation_info.cgi?id=4567) | [extracellular region](https://portal.genego.com/cgi/view_tree.cgi?type=loc&id=23&channel=Other&section=Network%20Statistics) | Receptor ligand |
| 59 | [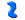](https://portal.genego.com/cgi/regulation/regulation_info.cgi?id=1172) | [Ubiquitin](https://portal.genego.com/cgi/regulation/regulation_info.cgi?id=1172) | [cytoplasm](https://portal.genego.com/cgi/view_tree.cgi?type=loc&id=1&channel=Other&section=Network%20Statistics) | Genetic binding protein |
| 60 | [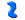](https://portal.genego.com/cgi/regulation/regulation_info.cgi?id=-1131633142) | [VE-cadherin](https://portal.genego.com/cgi/regulation/regulation_info.cgi?id=-1131633142) | [membrane](https://portal.genego.com/cgi/view_tree.cgi?type=loc&id=8&channel=Other&section=Network%20Statistics) | Genetic binding protein |
| 61 | [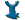](https://portal.genego.com/cgi/regulation/regulation_info.cgi?id=811) | [VEGFR-2](https://portal.genego.com/cgi/regulation/regulation_info.cgi?id=811) | [membrane](https://portal.genego.com/cgi/view_tree.cgi?type=loc&id=8&channel=Other&section=Network%20Statistics) |  |
| 62 | [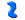](https://portal.genego.com/cgi/regulation/regulation_info.cgi?id=2662) | [VIL2 (ezrin)](https://portal.genego.com/cgi/regulation/regulation_info.cgi?id=2662) | [cytoplasm](https://portal.genego.com/cgi/view_tree.cgi?type=loc&id=1&channel=Other&section=Network%20Statistics) | Genetic binding protein |
| 63 | [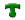](https://portal.genego.com/cgi/regulation/regulation_info.cgi?id=818) | [WNT](https://portal.genego.com/cgi/regulation/regulation_info.cgi?id=818) | [extracellular region](https://portal.genego.com/cgi/view_tree.cgi?type=loc&id=23&channel=Other&section=Network%20Statistics) | Receptor ligand |
